# Remarkable Homeostasis of Protein Sialylation in Skeletal Muscles of Hibernating Daurian Ground Squirrels (*Spermophilus dauricus*)

**DOI:** 10.3389/fphys.2020.00037

**Published:** 2020-02-07

**Authors:** Kai Dang, Han-Jie Yu, Shen-Hui Xu, Tian-Ran Ma, Hui-Ping Wang, Yang Li, Zheng Li, Yun-Fang Gao

**Affiliations:** ^1^Key Laboratory of Resource Biology and Biotechnology in Western China, Ministry of Education, Northwest University, Xi’an, China; ^2^Laboratory for Bone Metabolism, Key Laboratory for Space Bioscience and Biotechnology, School of Life Sciences, Northwestern Polytechnical University, Xi’an, China; ^3^Laboratory for Functional Glycomics, College of Life Sciences, Northwest University, Xi’an, China

**Keywords:** disuse muscle atrophy, hibernation, glycoproteomics, protein glycosylation, protein sialylation, interbout arousal, Daurian ground squirrel, soleus muscle

## Abstract

As the most common post-translational protein modification, glycosylation is intimately linked to muscle atrophy. This study aimed to investigate the performance of protein glycosylation in the soleus muscle (SOL) in Daurian ground squirrels (*Spermophilus dauricus*) and to determine the potential role of protein glycosylation in the mechanism underlying disuse muscle atrophy prevention. The results showed that (1) seven glycan structures comprising sialic acid α2-3 galactose (SAα2-3Gal) were altered during hibernation; (2) alterations in the SAα2-3Gal structure during hibernation were based on changes in the expression levels of beta-galactoside alpha-2 and 3-sialyltransferases; and (3) α2-3–linked sialylated modifications of heat shock cognate 70 and pyruvate kinase and expression of 14-3-3 epsilon protein were oscillatorily changed during hibernation. Our findings indicate that the skeletal muscles of hibernating Daurian ground squirrels maintain protein sialylation homeostasis by restoring sialylation modification during periodic interbout arousal, which might protect the skeletal muscles against disuse atrophy.

## Introduction

Since skeletal muscles control mobility, they have a significant impact on overall health and well-being. Skeletal muscle atrophy leads to deterioration in muscle function and increases fatigability and susceptibility to pathological and stress fractures (Degens and Alway, 2006; Bonaldo and Sandri, 2013). Muscle disuse and physical inactivity are common causes of muscle atrophy (Bodine, 2013a; Rudrappa et al., 2016; Gao et al., 2018). Although disuse muscle atrophy is a common clinical problem, there is currently no effective treatment option available to prevent disease progression. This is primarily due to a poor understanding of the cellular and molecular mechanisms underlying disuse muscle atrophy.

Approximately 70% of all human proteins are modified by diverse glycan structures, making glycosylation the most common post-translational protein modification (Apweiler et al., 1999). Glycans can enhance protein stability via nascent peptide chain folding, protect proteins from protease digestion, modify protein–protein binding and enzymatic activity, control protein secretion events, and mediate intercellular interaction and signaling (Haltiwanger, 2002; Haltiwanger and Lowe, 2004; Helenius and Aebi, 2004). Glycosylation, such as O-linked *N*-acetylglucosaminylation (*O*-GlcNAc), has been implicated in the regulation of disuse muscle atrophy (Cieniewski-Bernard et al., 2004, 2006). Another glycan modification–protein sialylation–has been reported to be associated with aging-related fiber degeneration and loss of muscle strength and mass, and sialic acid has been considered a biomarker for aging-related changes in muscles (Cieniewski-Bernard et al., 2006; Marini et al., 2014). Moreover, abnormal protein glycosylation has been shown to be involved in various muscle diseases, such as hereditary inclusion body myopathy, distal myopathy with rimmed vacuoles, and congenital muscular dystrophy (Martin-Rendon and Blake, 2003; Haliloglu and Topaloglu, 2004; Malicdan et al., 2008; Stalnaker et al., 2011). Lectins recognize and bind to specific glycan epitopes of glycoproteins. Large-scale characterization and quantitative analysis of glycans and glycoproteins can be achieved using a lectin microarray (Tateno et al., 2007; Ribeiro and Mahal, 2013) or another strategy involving lectin-based enrichment of glycan structures coupled with mass spectrometry (MS) (Wu et al., 2012; Yang et al., 2013b).

Compared with non-hibernating animals, hibernators show minimal loss in skeletal muscle mass and less physiological impairment of skeletal muscle function during hibernation (Lohuis et al., 2007; Bodine, 2013b). Thus, we used Daurian ground squirrels (*Spermophilus dauricus*), which hibernate for 4–5 months every year, to investigate the relationship between alterations in glycosylation patterns during hibernation and muscle atrophy. Previous work has demonstrated that antigravity soleus muscle (SOL) atrophy was due to disuse in non-hibernators (Ohira et al., 2002; Gustafsson et al., 2010). Given the close correlation between protein glycosylation and disuse muscle atrophy in non-hibernators, the absence of atrophy in the SOL muscle in Daurian ground squirrels during hibernation led to the speculation that hibernators could exhibit alterations in their glycosylation expression profile that could potentially prevent disuse muscle atrophy (Gao et al., 2012; Dang et al., 2016a, b). Thus, we performed this follow-up study to investigate the underlying protective mechanism utilized by SOL muscles of Daurian ground squirrels during hibernation using lectin microarray coupled with MS and bioinformatics analyses. This study provides further insight into the role of glycosylation in the skeletal muscles of Daurian ground squirrels during hibernation. Importantly, the results suggest the significance of homeostatic maintenance of glycosylation levels in skeletal muscles in preventing disuse muscle atrophy in hibernators.

## Materials and Methods

### Animals and Study Groups

The Daurian ground squirrels were collected and cared for as described previously (Gao et al., 2012; Dang et al., 2016b). Briefly, adult animals of both sexes were captured in Wei Nan, Shaanxi Province, China, in late August 2013. The animals were transported to Northwest University in Xi’an and housed individually in plastic cages (0.55 × 0.4 × 0.2 m^3^) in a colony room with an ambient temperature of 18–25°C and a 12-h:12-h light:dark cycle. The animals were supplied with standard rodent chow and water *ad libitum*, supplemented with fresh fruit and vegetables. After 1–2 months of acclimation, 32 animals (22 females, 10 males) were carefully weight matched and assigned randomly to the following four groups (*n* = 8/group): PRE, animals investigated in late autumn as the control, with body temperatures (Tbs) of 36–38°C; HIB, animals examined after 2 months hibernation with Tbs maintained at 5–8°C; IBA, animals examined while awake after 2 months hibernation with Tbs returned to 34–37°C for several hours; and POST, animals examined after waking from hibernation and maintaining Tbs of 36–38°C for more than 2 days.

**FIGURE 1:**
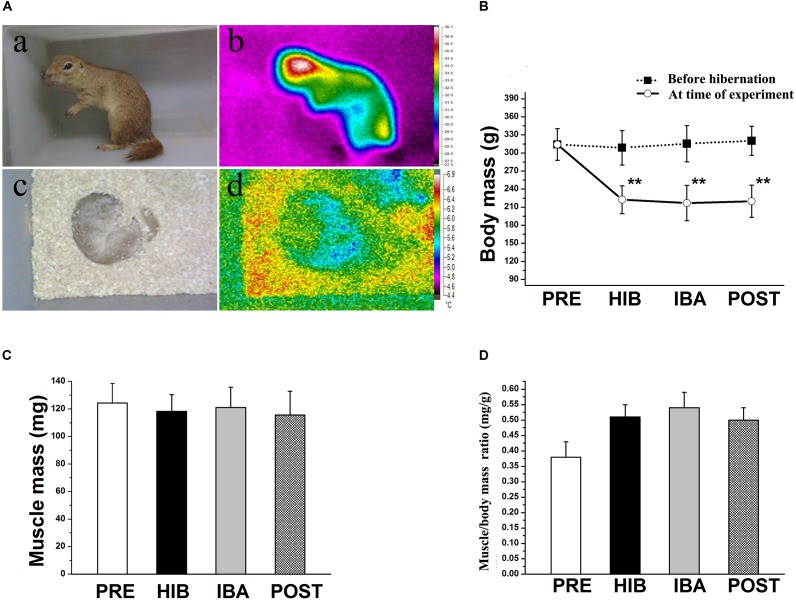
Changes in body temperature and soleus (SOL) muscle mass of Daurian ground squirrels during hibernation. **(A)** Body temperature was detected using a visual thermometer: **(a)** Photograph and **(b)** thermal image of a non-hibernating squirrel. **(c)** Photograph and **(d)** thermal image of a hibernating squirrel. **(B)** Changes in body mass of squirrels during different periods of hibernation. **(C)** Changes in SOL muscle wet mass of squirrels during different periods of hibernation. **(D)** Changes in SOL muscle/body mass ratio of squirrels during different periods of hibernation. Data are expressed as means ± standard deviations, *n* = 8. Analysis of variance was used to assess differences among groups. **P < 0.01 vs. PRE. PRE, pre-hibernation group; HIB, hibernation group; IBA, interbout arousal group; POST, post-hibernation group.

In late October 2013, the eight active animals in the PRE group were sacrificed. After presenting evidence of torpor, the remaining animals were transferred to a dark hibernaculum maintained at 4–6°C. Individual observation was performed, and Tbs were measured daily using a visual thermometer (Thermal Imager Ti125; Fluke Corporation, Everett, WA, United States) for the entire hibernation period. Hibernation was identified by low Tbs (5–8°C; Figure 1A), curling of the body, and torpor state. Recovery of Tbs and displacement of sawdust on the back were used to determine periodic arousal during hibernation. Eight animals experiencing 2 months hibernation were designated as the HIB group and were euthanized in the torpid hypothermic state. Animals that experienced at least 2 months hibernation and interbout arousal (IBA group) were euthanized in the early phase of arousal (2–3 h after onset). In April 2014, the remaining animals (POST group) naturally emerged from hibernation and were euthanized 2 days later. All animal handling and care protocols were approved by the Laboratory Animal Care Committee of China’s Ministry of Health. All experimental procedures were reviewed and pre-approved by the Northwest University Ethics Committee.

### Muscle Collection

All animals were anesthetized with sodium pentobarbital (90 mg/kg i.p.) prior to sacrifice. The SOL muscles were obtained from both hindlimbs of each animal, and muscle wet mass was recorded. The left SOL muscles were prepared for histochemical analysis, and the right muscles were flash frozen in liquid nitrogen and stored at −70°C until further processing.

### Immunohistochemical Analysis

Transverse sections (10 μm) were cut from the mid-belly of each SOL muscle at −20°C with a cryostat (CM1850; Leica, Wetzlar, Germany). Immunohistochemical analysis was used to determine muscle fiber CSA and distribution. After being air dried for 10 min and fixed in 4% paraformaldehyde in phosphate-buffered saline (PBS, pH 7.4) for 20 min, the sections were incubated in 5% bovine serum albumin (BSA; Boster, Wuhan, China) for 30 min at room temperature and then incubated in anti-skeletal fast myosin antibody (Sigma-Aldrich, St. Louis, MO, United States) at 4°C overnight. Subsequently, sections were washed (4 × 15 min) in PBS with 1% BSA and then incubated in anti-mouse polyvalent immunoglobulin (G,A,M)-fluorescein isothiocyanate antibody produced in goat (diluted 1:1,000 in PBS with 1% BSA; Sigma-Aldrich) for 2 h at 37°C to bind to the primary antibody. Sections were washed (4 × 15 min) in PBS with 1% BSA, then lightly coated with ProLong Gold antifade mountant (Thermo Fisher Scientific, San Jose, CA, United States). The sections were imaged using a laser scanning confocal microscope (LSCM; Nikon, Tokyo, Japan). A minimum of three fibers or 600 cells per sample was counted.

The SOL muscle fiber distribution and CSA analyses were carried out as described previously (Gao et al., 2012). Briefly, muscle type was established from at least 300 fibers per muscle. Fiber distribution was expressed as the number of fibers of each type relative to the total number of fibers. The CSA of at least 100 fibers per muscle was measured using a Quantimet-570 video image analyzer (Leica Microsystems Ltd., London, United Kingdom).

### Total Protein Extraction

Total proteins were extracted from the SOL muscles using RIPA lysis buffer (Beyotime Institute of Biotechnology, Haimen, China) with a protease inhibitor tablet cocktail (Pierce Chemical Co., Rockford, IL, United States) according to the manufacturer’s guidelines. Briefly, frozen samples were homogenized in RIPA lysis buffer with a protease inhibitor (10 μL mg^–1^) and then incubated on ice for 30 min. Following centrifugation (10,000 × *g*, 10 min, 4°C), the supernatant was collected and transferred for use or stored at −70°C. The bicinchoninic acid assay (Pierce Chemical Co.) was carried out to determine the protein concentration.

### Lectin Microarray and Data Analysis

The lectin microarray was performed as described previously (Qin et al., 2012; Yu et al., 2012). Briefly, 37 lectins obtained from Vector Laboratories (Burlingame, CA, United States), Sigma-Aldrich, and Calbiochem Merck (Darmstadt, Germany) were dissolved in the manufacturer-recommended buffer to a final concentration of 1 mg/mL, and then printed on homemade epoxysilane-coated slides with Stealth Micro spotting pins (SMP-10B; TeleChem, Atlanta, GA, United States) using a Capital smart microarrayer (CapitalBio, Beijing, China). Each lectin was printed in triplicate per block, with triplicate blocks placed on one slide. The glycan-binding specificities of the lectins and layout of the lectin microarrays are shown in Additional Files 4 and 5 (Supplementary Table S1 and Supplementary Figure S1).

The extracted muscle proteins were labeled with Cy3 fluorescent dye and purified using a Sephadex-G25 column (GE Healthcare, Buckinghamshire, United Kingdom). The Cy3-labeled proteins were then applied to the lectin microarrays. Incubation was performed at 37°C for 3 h in a rotisserie oven set at 4 rpm. After incubation, the slides were washed three times in PBS with Tween 20 (PBST) for 5 min each time and once in PBS for 5 min, then dried by centrifugation at 600 rpm for 5 min at room temperature. The microarrays were finally scanned at 70% photomultiplier tube and 100% laser power settings with a Genepix 4000B confocal scanner (Axon Instruments, Inc., Sunnyvale, CA, United States). The acquired images and signal intensities were analyzed using Genepix 3.0 software (Axon Instruments Inc., Foster City, CA, United States) under Cy3 mode (532 nm). The net fluorescence intensity for each spot was calculated by subtracting the background intensity. Values less than the average background ± 2 standard deviations (SDs) were removed. Each protein sample was technically repeated for nine blocks (three slides). The median fluorescence intensity for each lectin was normalized to the sum of the medians in one block. The normalized median and SD for each lectin were averaged from nine repeated blocks. The normalized data were compared among groups to identify relative changes in protein glycosylation levels. Hierarchical clustering was performed with EXPANDER software (v6.0)^1^. One-way analysis of variance (ANOVA) was used to identify significant differences (SPSS v10.0; SPSS Inc., Chicago, IL, United States).

### Lectin Histochemical Analysis

Skeletal muscle specimens from the four groups of squirrels were subjected to lectin histochemical analysis as described previously (Qin et al., 2012), with some modifications. Briefly, frozen sections (10 μm) were mounted on glass slides and dried at room temperature. Fixation was achieved by incubation with 4% paraformaldehyde in PBS for 10 min at room temperature. Subsequently, the sections were blocked with blocking buffer (5% BSA in PBS) at room temperature for 1 h. The sections were then stained with Cy3 fluorescein-labeled lectins to a final concentration of 20 μg/mL at 4°C in the dark overnight, then washed three times with PBS for 10 min each time. The sections were incubated with 2 μg/mL DAPI (Sigma-Aldrich) for 10 min to label the nuclei, and then washed with PBS. Finally, image acquisition and fluorescence scanning were performed with an LSCM (Nikon).

### Real-Time Polymerase Chain Reaction (PCR)

Total RNA was isolated from the SOL muscles using RNAiso Plus reagent (TaKaRa Bio, Dalian, China) according to the manufacturer’s protocol. RNA sample integrity was examined using 1% agarose gel electrophoresis. Nanophotometry (IMPLEN, Munich, Germany) was applied to confirm the concentration and purity of RNA samples, with samples showing 260/280 optical densities of 1.8–2.0 deemed to be acceptable. Total RNA was reverse transcribed into cDNA using the PrimeScript RT Master Mix (TaKaRa Bio, Japan). All primers were designed using PrimerQuest^2^ to produce exon-spanning amplicons of 70–150 bp and purchased from Generay Biotech Co., Ltd. (Shanghai, China). All primers were tested for efficiency by serial dilution of cDNAs. The efficiencies of the target and reference gene amplifications were 90–110% and approximately equal. Real-time PCR was performed using a LightCycler 480 (Roche Applied Science, Mannheim, Germany) and 20 ng total RNA as the template. The PCR mixture included 2 μL diluted single-stranded cDNA, 1.6 μL gene-specific primer set (0.8 μM), 10 μL SYBR Premix Ex TaqII (2×; TaKaRa), and 6.4 μL sterile distilled water in a final volume of 20 μL. The PCR thermocycling protocol comprised initial denaturation at 95°C for 5 min, followed by 40 cycles of 30 s at 95°C and 40 s at 58°C. All PCR assays were performed in triplicate. PCR product melting curves displaying single sharp peaks confirmed the specificity of primer annealing (Supplementary Figure S2). To normalize the mRNA expression of each target gene, GAPDH was used as the housekeeping gene. The relative expression of all genes was determined using the 2^–ΔΔ*CT*^ equation. The gene-specific primers used are listed in Table 1.

**TABLE 1:** List of primers used for qRT-PCR.

**Primer**	**Forward**	**Reverse**
ST3Gal1	5′-ATGGTCCTCGTGCCC TTCA-3′	5′-GTCCTGCTTCACCTTGATC TTCG-3′
ST3Gal2	5′-TCTACAACCCAGCCTT CTTCA-3′	5′-CGTTCACCTCATCACA CACA-3′
ST3Gal3	5′-GCCTGAACAGTATGAGC GTGAC-3′	5′-GCCACCGATTTCCAG AAGC-3′
ST3Gal5	5′-TCACGCTACTGACCTGTTT GTTG-3′	5′-TTTTCTGCCACCTGCT TCCA-3′
Neu1	5′-GACCTTTGACCCTGAA CTGG-3′	5′-GAACTCTGGATGGGCT GGAT-3′
Neu3	5′-CACTGGTCTCACCCTT GGAT-3′	5′-ACTCGGTCCCACACTC AAAC-3′
GAPDH	5′-GACAACTTTGGCATCG TGGA-3′	5′-ATGCAGGGATGATGTT CTGG-3′

*ST3Gal1–6: beta-galactoside alpha-2 and 3-sialyltransferases 1–6; NEU1–4: sialidases.*

### Selective Isolation of Glycoproteins

Selective isolation of glycoproteins was performed according to previously established procedures (Yang et al., 2012, 2013a,b). In brief, total muscle proteins (2 mg) extracted from each group were diluted to 600 μL with binding buffer (0.1 M Tris-HCl, 150 mM NaCl, 1 mM CaCl_2_, 1 mM MgCl_2_, and 1 mM MnCl_2_; pH 7.4) containing 6 μL of a proteinase inhibitor cocktail (Sigma-Aldrich). Homemade MAL-II magnetic particles were rinsed with binding buffer and then incubated in the diluted protein solution at room temperature for 1 h under gentle shaking. After washing three times with washing buffer (binding buffer with 0.1% Tween 20), the unbound proteins were removed. The captured glycoproteins were eluted with 300 μL elution buffer (0.5 M methyl-α-D-mannopyranoside in binding buffer supplemented with 3 μL proteinase inhibitor cocktail). The eluted glycoproteins were denatured with 8 M urea at room temperature for 30 min.

### Glycoprotein Digestion and Identification of Peptides by LC-ESI-MS/MS

The pH of the glycoprotein solution was adjusted to 8.5 with 1 M ammonium bicarbonate. After chemical reduction with the addition of 10 mM *dithiothreitol*, the glycoproteins were carboxyamidomethylated by treatment with 55 mM iodoacetamide, and then digested with Trypsin Gold (Promega, Madison, WI, United States) to a final substrate/enzyme ratio of 30:1 (w/w) at 37°C for 16 h. After digestion, the tryptic peptides were acidified with 10 μL formic acid and desalted with a Strata X column (Phenomenex, Los Angeles, CA, United States). The resulting peptides were dried in a speed vacuum and resolubilized in 200 μL buffer A [2% acetonitrile (ACN), 0.1% formic acid (FA)]. Following centrifugation at 20,000 × *g* for 10 min at room temperature, a peptide solution with a final concentration of ∼0.5 μg/μL was obtained for LC-ESI-MS/MS analysis with the Triple TOF 5600 system (AB SCIEX, Concord, ON, Canada).

Peptide separation was performed with an LC-20AD nano–high-performance liquid chromatograph (Shimadzu, Kyoto, Japan). The peptide solution (10 μL) was loaded onto a 2-cm C18 trap column, and then eluted onto a 10-cm analytical C18 column (inner diameter, 75 μm). The samples were loaded at a rate of 8 μL/min for 4 min. The linear gradient steps for peptide separation were as follows: 35 min gradient at 300 nL/min from 2% to 35% B (95% ACN, 0.1% FA), 5 min linear gradient to 60%, 2 min linear gradient to 80%, maintenance at 80% B for 4 min, and return to 5% in 1 min.

Data were acquired using the Triple TOF 5600 system equipped with a Nanospray III source (AB SCIEX) and a pulled quartz tip (New Objectives, Woburn, MA, United States). An ion spray voltage of 2.5 kV, curtain gas of 30 psi, nebulizer gas of 15 psi, and interface heater temperature of 150°C were set. The system was operated with a rotary pump of no less than 30,000 full-width half-maximum for the time-of-flight MS scans. For information-dependent acquisition, survey scans with 2+ to 5+ charge states were obtained at 100 ms, and 40 product ion scans beyond 150 counts/s were collected. The Q2 transmission window was set at 80 and 100 Da for 50%.

### Data Analysis

Raw MS files were converted to MGF files with Proteome Discoverer 1.2 (Thermo Fisher Scientific) and then searched using the Mascot search engine (v2.3.02; Matrix Science, London, United Kingdom) against the most recently updated Rodentia Uniprot database (30 April 2015; 265,553 entries).

The search parameters were set as follows: mass tolerances of 0.05 Da for peptides and 0.1 Da for fragmented ions; max trypsin missed cleavage of 1; Gln- > pyro-Glu (N-term Q), oxidation (M), and deamidated (NQ) as the potential variable modifications; and carbamidomethyl (C) as the fixed modification. A decoy database search was performed in Mascot. Peptides with significance scores ≥ 20 at the 99% confidence level, as determined by Mascot probability analysis, were counted as identified.

### Analysis of Identified Protein Bioinformatics

GO annotations of the identified proteins were obtained using the Blast2GO program (Gotz et al., 2008). Kyoto Encyclopedia of Genes and Genomes (KEGG) pathway enrichment analysis of the identified proteins was performed using Fisher’s hypergeometric test (Lu et al., 2014; Lv et al., 2015) with a threshold of *P* < 0.05. Protein–protein interaction (PPI) analysis of the identified proteins from two groups was performed using the IntAct molecular interaction database and visualized by construction of a PPI network using Cytoscape software (Kohl et al., 2011).

### Western Blotting

An equal amount of protein (40 μg) from each SOL muscle sample was separated by sodium dodecyl sulphate polyacrylamide gel electrophoresis (SDS-PAGE), and then transferred onto polyvinylidene fluoride membranes (Millipore, Bedford, MA, United States) using a semi-dry transfer apparatus (Bio-Rad Laboratories, Hercules, CA, United States). Membranes were blocked with 5% skim milk dissolved in Tris-buffered saline with Tween 20 (TBST pH 7.6; 10 mM Tris-HCl, 150 mM NaCl, 0.05% Tween20) for 2 h at room temperature, followed by overnight incubation with appropriate primary antibodies, including rabbit monoclonal anti–14-3-3 epsilon (Abcam, Cambridge, MA, United States), rabbit monoclonal anti-HSC70 (Abcam), rabbit monoclonal anti–PK isozymes M1/M2 (Cell Signaling Technology, Beverly, MA, United States), and rabbit polyclonal anti-GAPDH (Santa Cruz Biotechnology, Santa Cruz, CA, United States). All antibodies were diluted to the recommended concentrations with TBST. Membranes were washed three times with TBST for 15 min each time, and then incubated with horseradish peroxidase–conjugated anti-rabbit secondary antibodies (Pierce Chemical Co.) for 1 h at 37°C. Following washing with TBST (3 × 15 min), immunoblots were visualized using enhanced chemiluminescence reagents (Pierce Chemical Co.). Quantity One software (Bio-Rad Laboratories) was used for quantification. The expression of GAPDH was used as an internal control.

### Lectin/Glyco-Antibody Microarrays

Lectin/glyco-antibody microarrays were carried out as described previously (Li et al., 2009; Yang et al., 2013b). Briefly, antibodies were spotted on homemade epoxysilane-coated slides with Stealth Micro spotting pins (SMP-10B; TeleChem) using a Capital smart microarrayer (CapitalBio, Beijing, China). Each antibody was printed in triplicate in one block. The slides were incubated in a humidity-controlled incubator (at 50% humidity) overnight to immobilize antibodies. After washing with coupling buffer supplemented with 0.05% Tween 20 (0.02 M sodium acetate; pH 5.5) and coupling buffer alone (0.02 M sodium acetate; pH 5.5), the printed slides were oxidized with 200 mM NaIO_4_ solution at room temperature (18–22°C) in the dark for 30 min, then rinsed three times with coupling buffer. Subsequently, the slides were immersed in 1 mM 4-hydroxybenzhydrazide in dimethylformamide at room temperature for 2 h to allow derivatization of the carbonyl groups. The slides were blocked with 1 × Carbo-free blocking solution (Vector Laboratories) diluted with PBST for 1 h, and then dried by centrifugation at 600 rpm for 5 min at room temperature. Afterward, 120 μL total proteins from each SOL sample (diluted with 1 × Carbo-free blocking solution) was applied to the antibody microarrays and incubated in a humidified chamber at 37°C for 3 h. To remove unbound proteins, the slides were rinsed three times with PBST for 5 min each time, followed by a 5-min wash with PBS. The slides were treated with Cy3-labeled MAL-II in diluted 1 × Carbo-free blocking solution in a humidified chamber at 37°C for 3 h, and then washed with PBS and dried by centrifugation at 600 rpm for 5 min. Finally, the slides were scanned with a Genepix 4000B microarray scanner and analyzed using GenePix 3.0 software (Axon Instruments Inc.). The average background was subtracted, and values less than the average background ± 2 SDs were removed. The data points for each antibody were multiplied by a normalization factor derived from the ratio of the sum of the medians of data points in one block to that of data points in another block. Each sample was technically repeated for three slides, and the normalized value of each antibody was averaged from six repeated blocks.

### Statistical Analysis

All data were analyzed using SPSS software v10.0 (SPSS Inc., Chicago, IL, United States) and are expressed as mean ± SD. One-way ANOVA with Tukey’s *post hoc* tests were used to identify significant differences among groups. Statistical significance was set at *P* < 0.05.

## Results

### SOL Muscle Size and Morphology Do Not Change in Daurian Ground Squirrels During Hibernation

**FIGURE 2:**
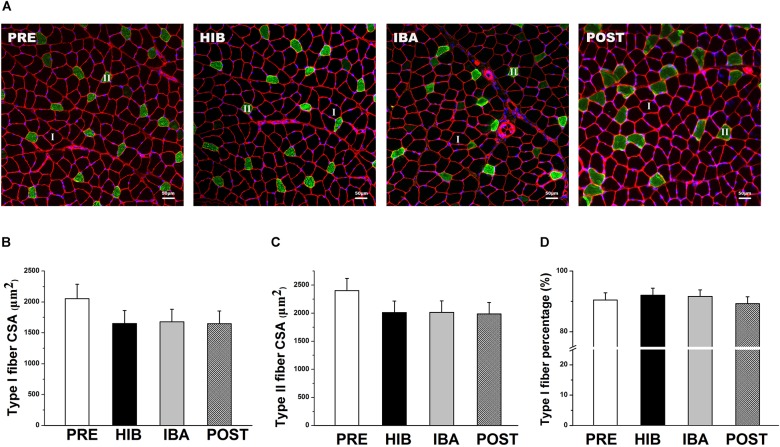
Soleus (SOL) muscle size and morphology did not change during hibernation in Daurian ground squirrels. **(A)** Representative immunohistochemical images of the SOL muscles of squirrels during different periods of hibernation. I = type I fiber; II = type II fiber. **(B)** Changes in the type I fiber cross-sectional area (CSA) of squirrel SOL muscles during different periods of hibernation. **(C)** Changes in the type II fiber CSA of squirrel SOL muscles during different periods of hibernation. **(D)** Percentages of type I fiber in squirrel SOL muscles during different periods of hibernation. Data are expressed as means ± standard deviations, *n* = 8. Analysis of variance was used to assess differences among groups. PRE, pre-hibernation group; HIB, hibernation group; IBA, interbout arousal group; POST, post-hibernation group.

Significant decreases in ground squirrel body weight were observed in the hibernation (HIB; 29.65%), interbout arousal (IBA; 31.87%), and post-hibernation (POST; 30.58%) groups compared with the pre-hibernation (PRE) group (314.2 g; all *P* < 0.01). However, no significant difference was observed in the SOL muscle wet weight or muscle/body mass ratio among the four groups (Figures 1B–D). Immunohistochemical analysis was used to determine SOL muscle fiber types (Figure 2A), and no significant difference in the SOL muscle fiber cross-sectional area (CSA) or distribution was found among groups (Figures 2B–D). Thus, the SOL muscle size and morphology remained unchanged in Daurian ground squirrels during prolonged periods of hibernation inactivity and nutritional deficiency.

### Glycopatterns in the SOL Muscles of Daurian Ground Squirrels Differ Among Hibernation Periods, as Determined by Lectin Microarray

**FIGURE 3:**
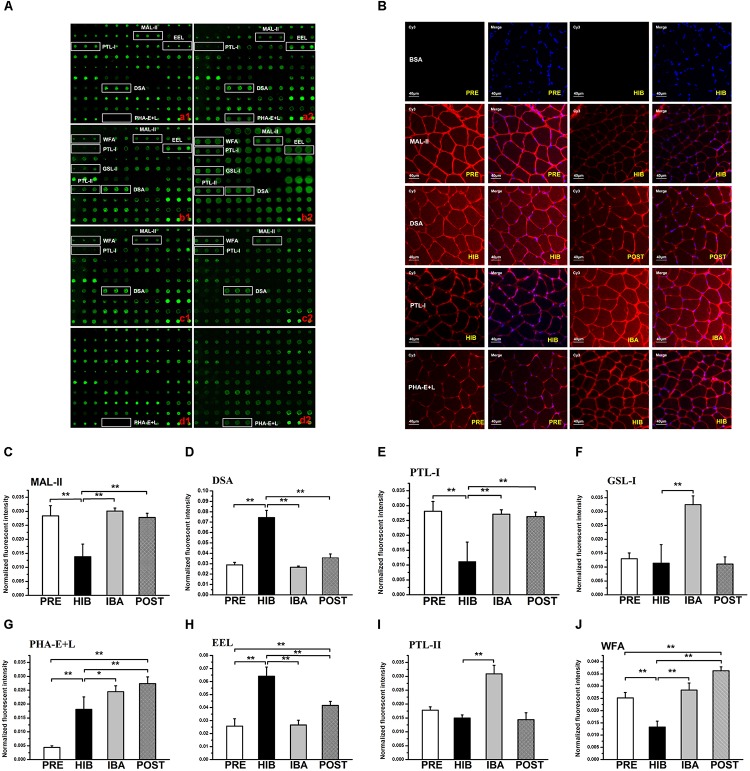
Changes in protein glycosylation in Daurian ground squirrel soleus (SOL) muscles during hibernation. **(A)** Glycan profiling of SOL muscles in squirrels during different periods of hibernation using lectin microarray. Lectins showing significant differences in glycan expression are marked; **(a1,a2)**: analysis of PRE **(a1)** and HIB **(a2)** groups; **(b1,b2)**: analysis of HIB **(b1)** and IBA **(b2)** groups; **(c1,c2)**: analysis of HIB **(c1)** and POST **(c2)** groups; **(d1,d2)**: analysis of PRE **(d1)** and POST **(d2)** groups. **(B)** Validation by lectin histochemistry. Frozen SOL sections were double stained with Cy3-labeled lectins and DAPI. Results were consistent with the microarray results. **(C–J)** Normalized fluorescence intensity of lectins with altered signals in the lectin microarray (ratio > 2.0 or <0.5). Analysis of variance was used to assess differences among groups. *P < 0.05, **P < 0.01. MAL-II, *Maackia amurensis* lectin II; PTL*-I*, *Psophocarpus tetragonolobus lectin I; EEL, Euonymus europaeus lectin;* DSA, *Datura stramonium* agglutinin; PHA-E + L, *Phaseolus vulgaris* lectin; WFA, *Wisteria floribunda lectin; GSL-I, Griffonia simplicafolia* lectin I; PTL*-II*, *Psophocarpus tetragonolobus lectin II.* PRE, pre-hibernation group; HIB, hibernation group; IBA, interbout arousal group; POST, post-hibernation group.

The lectin microarray results revealed altered protein glycopatterns in the SOL muscles of Daurian ground squirrels during different periods of hibernation; lectins showing differences in glycan expression among groups are framed in white in Figure 3A. Differential analysis revealed obvious differences (ratio > 2.0 or < 0.5; *P* < 0.05) in specific lectins among groups, indicating significant alterations in the abundance of glycans recognized by different lectins (Table 2 and Figures 3C–J). Results showed that (1) the level of sialic acid α2-3 galactose (SAα2-3Gal), recognized by MAL-II, was significantly decreased in the HIB group compared with the PRE group, but was significantly increased and recovered to pre-hibernation levels in the IBA and POST groups; (2) the level of *N*-acetylglucosamine (GlcNAc), recognized by *Datura stramonium* agglutinin (DSA), was significantly increased in the HIB group compared with the PRE group, but was significantly increased and recovered to pre-hibernation levels in the IBA and POST groups; (3) changes in the levels of α *N*-acetylgalactosamine (αGalNAc) and Gal, recognized by *Psophocarpus tetragonolobus lectin (PTL)* I, were similar to that of SAα2-3Gal, i.e., levels were decreased in the HIB group but increased in the IBA and POST groups; (4) the level of Galα1-3 (Fucα1-2) Gal, recognized by *Euonymus europaeus lectin*, was increased in the HIB group compared with the other three groups; (5) the levels of αGalNAc and Gal, recognized by *Griffonia simplicifolia* lectin I (GSL-I), were significantly increased in the IBA group compared with the other three groups; (6) the levels of tri- and tetra-antennary complex-type *N*-glycans, recognized by *Phaseolus vulgaris* lectin (PHA-E + L), were significantly increased in the HIB, IBA, and POST groups compared with the PRE group; (7) the levels of αGalNAc and Gal, recognized by PTL-II, were significantly increased in the IBA group compared with the other three groups; and (8) the level of terminal GalNAc, recognized by *Wisteria floribunda lectin*, was significantly decreased in the HIB group compared with the PRE group, and increased in the IBA and POST groups compared with the HIB group.

**TABLE 2:** Differential glycopatterns in the SOL muscles of hibernating Daurian ground squirrels, determined by lectin microarray analysis.

**Lectin**	**Specificity**	**HIB/PRE ratio**	**IBA/HIB ratio**	**POST/HIB ratio**	**POST/PRE ratio**
MAL-II	SAα2-3Gal	0.49	2.17	2.01	/
PTL-I	αGalNAc and Gal	0.40	2.43	2.35	/
WFA	Terminal GalNAc	/	2.14	2.73	/
GSL-I	αGalNAc	/	2.84	/	/
PTL-II	Gal	/	2.06	/	/
DSA	GlcNAc	2.57	0.36	0.48	/
EEL	Galα1-3 (Fucα1-2) Gal	2.49	0.41	/	/
PHA-E + L	Tri-and tetra- antennary complex-type *N*-glycans	4.08	/	/	6.22

*/ 0.5 ≤ ratio ≤ 2 (no difference between groups). SAα2-3Gal, sialic acid α2-3 galactose; Gal, galactose; GalNAc, *N*-acetylgalactosamine; GlcNAc, *N*-acetylglucosamine; Fuc, fucose.*

### Validation of Differential Glycopatterns by Lectin Histochemical Analysis

Four altered lectins (MAL-II, DSA, PTL-I and PHA-E + L) were selected and used to validate the microarray results using lectin histochemistry. Lectin staining of tissue sections (Figure 3B) showed that the fluorescence signal intensity of MAL-II was significantly decreased in the HIB group compared with the PRE group, whereas the intensity of DSA was significantly decreased in the POST group compared with the HIB group. The fluorescence signal intensity of PTL-I was significantly increased in the IBA group compared with the HIB group, and the intensity of PHA-E + L was significantly increased in the HIB group compared with the PRE group (all *P* < 0.05). Thus, the lectin histochemical results were comparable to the microarray results.

### mRNA Expression of Sialyltransferases and Sialidases in Different Periods of Hibernation

**FIGURE 4:**
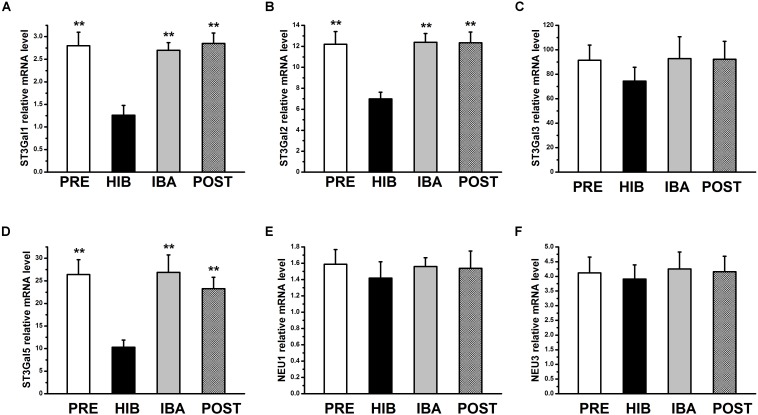
Relative mRNA expression of sialyltransferases and sialidases in soleus (SOL) muscles of hibernating Daurian ground squirrels. Relative mRNA levels of 3-sialyltransferases 1–6 (ST3Gal1–6) and sialidases (NEU1–4) in the SOL muscles of squirrels at different timepoints during hibernation. mRNA expression of ST3Gal1 **(A)**, ST3Gal2 **(B)** and ST3Gal5 **(D)** was changed and mRNA expression of ST3Gal3 **(C)**, NEU1 **(E)** and NEU3 **(F)** did not differ in the SOL muscles of squirrels during hibernation. mRNA levels of ST3Gal4, ST3Gal6, NEU2 and NEU4 in the SOL muscles of squirrels were below the detection levels. Data are expressed as means ± standard deviations, *n* = 8. Analysis of variance was used to assess differences among groups. **P < 0.01 vs. HIB. PRE, pre-hibernation group; HIB, hibernation group; IBA, interbout arousal group; POST, post-hibernation group.

MAL-II–binding Sia2-3Gal expression was significantly altered in different periods of hibernation. Therefore, we focused on sialyltransferases [beta-galactoside alpha-2 and 3-sialyltransferases 1–6 (ST3Gal1–6)] and sialidases (NEU1–4) for further experiments. Compared with the PRE group, the mRNA expression of ST3Gal1, ST3Gal2, and ST3Gal5 was significantly decreased by 55.00, 42.70, and 60.91%, respectively, in the HIB group (all *P* < 0.01), but returned to pre-hibernation levels in the IBA group (Figure 4). The mRNA expression of ST3Gal3 and NEU1,3 did not differ significantly among groups, and that of ST3Gal4, ST3Gal6, NEU2, and NEU4 was not detectable in any group, indicating extremely low levels (or absence) in the SOL muscles of Daurian ground squirrels.

### Identification and Properties of Glycoproteins With SAα2-3Gal Structure Isolated From the SOL Muscles of Daurian Ground Squirrels

Significant changes in the SAα2-3Gal structure specifically recognized by MAL-II in the SOL muscles of Daurian ground squirrels during hibernation may play an important protective role against disuse muscle atrophy. Therefore, we prepared lectin MAL-II magnetic microparticles, and then extracted glycoproteins with terminal SAα2-3Gal from the SOL muscles before and during hibernation. The extracted glycoproteins were identified by liquid chromatography–electrospray ionization–tandem mass spectrometry (LC-ESI-MS/MS), followed by a Uniprot database^3^ search. A total of 227 and 185 glycoproteins were identified in the PRE and HIB groups, respectively. There were 86 glycoproteins in common between these two groups (Supplementary Table S2).

**FIGURE 5:**
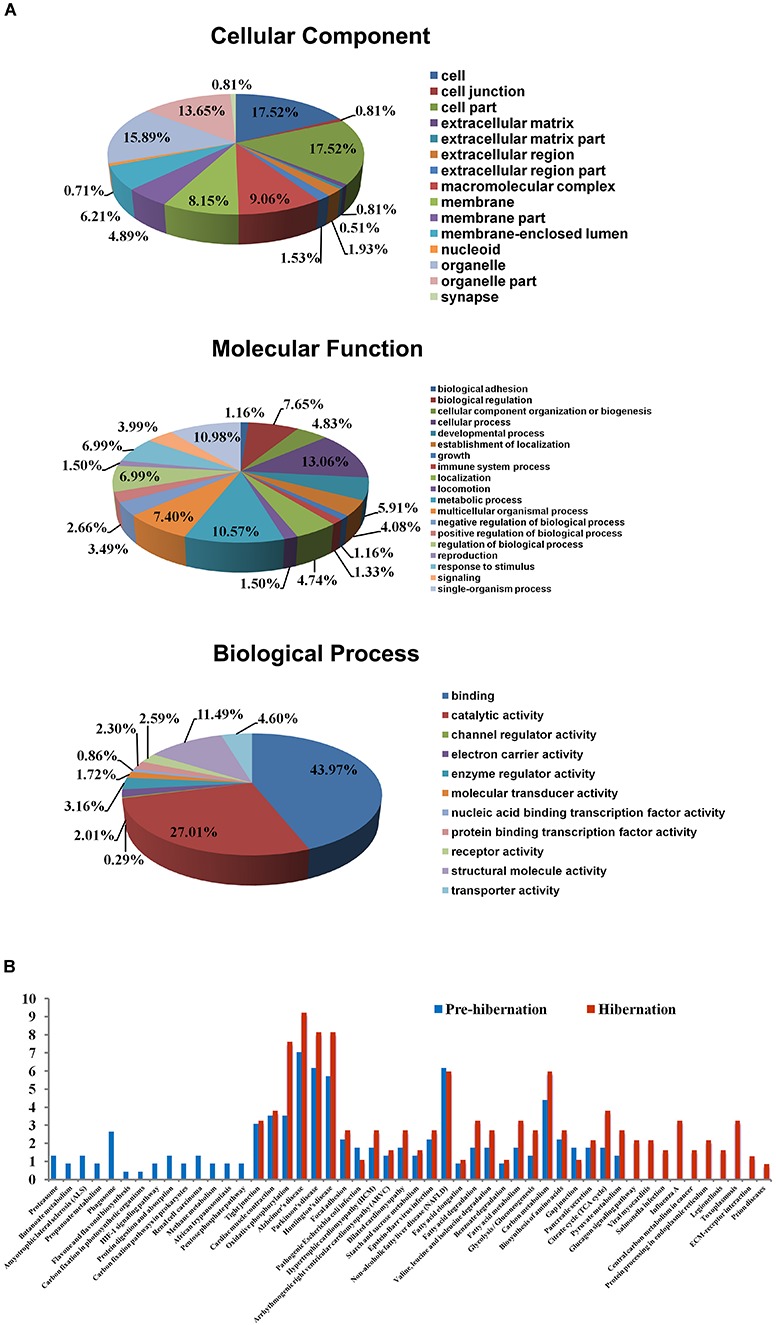
Bioinformatics analysis of glycoproteins with the sialic acid α2-3 galactose (SAα2-3Gal) structure from the soleus (SOL) muscles of hibernating Daurian ground squirrels. **(A)** Gene ontology annotation of all identified glycoproteins with the SAα2-3Gal structure in the SOL muscles of squirrels in the pre-hibernation (PRE) and hibernation (HIB) groups, conducted using Blast2GO. **(B)** Enrichment analysis of Kyoto Encyclopedia of Genes and Genomes pathways of the identified glycoproteins with the SAα2-3Gal structure in the SOL muscles of squirrels in the PRE and HIB groups, performed using Fisher’s hypergeometric test. Percentages of glycoproteins enriched in each pathway in the total isolated glycoprotein population are shown.

Blast2GO (Gotz et al., 2008) was used to analyze the function and distribution of the 326 glycoproteins identified in the PRE and HIB groups (Figure 5). According to cell component annotations (Figure 5A), the glycoproteins isolated by the MAL-II magnetic microparticles from the SOL muscles were located predominantly in the cell (17.52%), organelle (15.89%), and membrane (8.15%), and existed mainly as macromolecular complexes (9.06%). Based on molecular functional annotations (Figure 5A), the main functions of these glycoproteins were binding (43.97%), catalytic activity (27.01%), and structural molecule activity (11.49%). Based on biological process annotations (Figure 5A), the isolated glycoproteins were involved mainly in cellular processes (13.06%), single-organism processes (10.98%), metabolic processes (10.57%), and biological regulation (7.65%).

We analyzed protein enrichment for each KEGG pathway with all qualitative proteins serving as background using hypergeometric tests (*P* < 0.05), and further identified pathways with significant enrichment of the isolated glycoproteins. The results showed 40 and 36 pathways with significant enrichment of glycoproteins from the PRE and HIB groups, respectively (Supplementary Tables S3, S4), of which 14 were significantly enriched only before hibernation and 10 were significantly enriched only during hibernation (Figure 5B).

**FIGURE 6:**
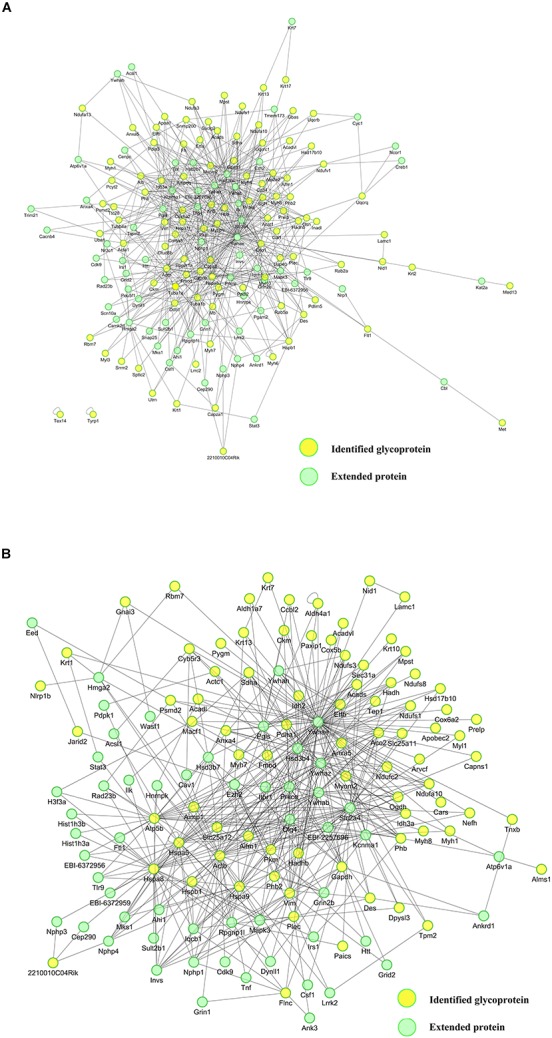
Protein–protein interaction networks of glycoproteins with the sialic acid α2-3 galactose (SAα2-3Gal) structure in the soleus (SOL) muscles of pre-hibernating **(A)** and hibernating **(B)** Daurian ground squirrels. Yellow circles represent glycoproteins identified in the present study; green circles represent extended proteins.

To systematically reveal the functions and PPIs of the glycoproteins bearing SAα2-3Gal extracted from the SOL muscles of Daurian ground squirrels and to explore the proteins potentially involved in the mechanism against disuse muscle atrophy, PPI analysis was performed. We used the IntAct database^4^ to construct glycoprotein PPI network maps for the PRE (Figure 6A) and HIB (Figure 6B) groups using Cytoscape software (Kohl et al., 2011; Zhang et al., 2015). We also calculated the degree of each node in the network, which indicates the number of links from a corresponding protein to all other proteins, with higher values signifying a greater number of links. Among all proteins, Ywhae (14-3-3 epsilon protein; *d* = 84) showed the highest degree value, suggesting close interaction with SAα2-3Gal–containing glycoproteins. Therefore, we hypothesized that the 14-3-3 epsilon protein plays a key role in the protective mechanism against disuse muscle atrophy.

### Changes in 14-3-3 Epsilon Protein Expression in the SOL Muscles of Daurian Ground Squirrels in Different Periods of Hibernation

**FIGURE 7:**
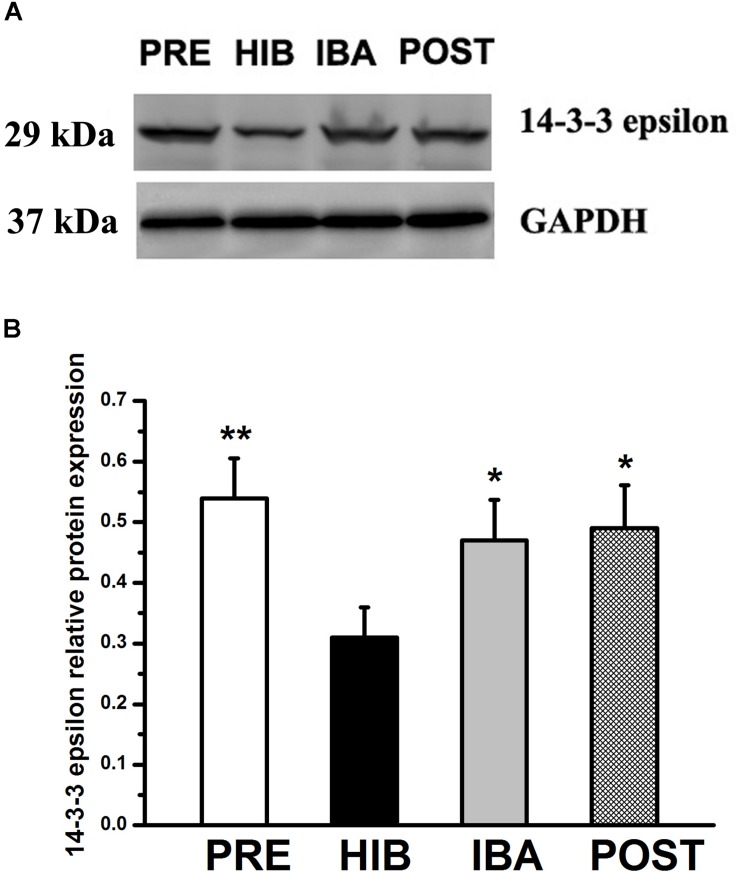
Changes in 14-3-3 epsilon protein expression in the soleus (SOL) muscles of Daurian ground squirrels during hibernation. **(A)** Representative western blots of 14-3-3 epsilon in the SOL muscles of squirrels. **(B)** Relative protein expression of 14-3-3 epsilon in the SOL muscles of squirrels during different periods of hibernation. Data are expressed as means ± standard deviations, *n* = 8. Analysis of variance was used to assess differences among groups. *P < 0.05, **P < 0.01 vs. HIB. PRE: pre-hibernation group; HIB, hibernation group; IBA, interbout arousal group; POST, post-hibernation group.

Given that the PPI analysis showed that 14-3-3 epsilon was the core protein in the glycoprotein interaction network, we investigated the expression of this protein during hibernation. Compared with the PRE group, 14-3-3 epsilon protein expression was decreased by 42.59% in the HIB group (*P* < 0.01). Compared with the HIB group, 14-3-3 epsilon protein expression increased significantly by 51.61 and 58.06% in the IBA and POST groups, respectively (both *P* < 0.01). No significant difference was found among the PRE, IBA, and POST groups (Figure 7).

### Validation by Western Blot and Lectin/Glyco-Antibody Microarray Analyses

Western blot and lectin/glyco-antibody microarray analyses were used to validate the glycoprotein extraction and MS results. We selected heat shock cognate 70 (HSC70) and pyruvate kinase (PK) for the validation.

**FIGURE 8:**
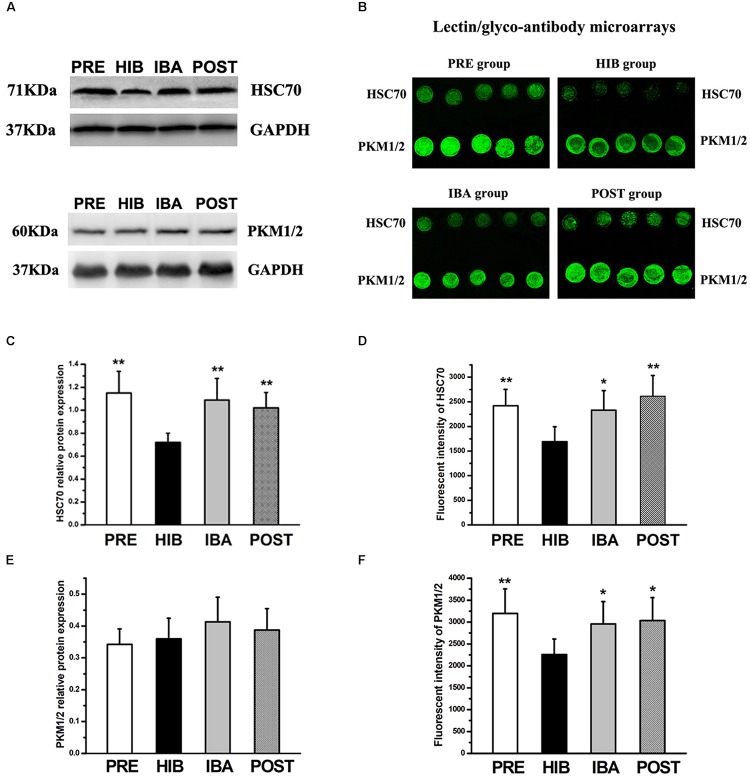
Expression changes of heat shock cognate 70 (HSC70) and pyruvate kinase isozymes M1/M2 (PKM1/2) in the soleus (SOL) muscles of hibernating Daurian ground squirrels. **(A)** Representative western blots of HSC70 and PKM1/2. **(B)** Images from lectin/glyco-antibody microarrays of HSC70 and PKM1/2. **(C,E)** Relative protein expression of HSC70 **(C)** and PKM1/2 **(E)** in the SOL muscles of squirrels during different periods of hibernation. **(D,F)** Analyses of lectin/glyco-antibody microarrays of HSC70 **(D)** and PKM1/2 **(F)**. *Maackia amurensis* lectin II was used in the microarrays, with weak fluorescence intensity of microarray signals representing low expression of the sialic acid α2-3 galactose structure. Data are expressed as means ± standard deviations, *n* = 8. Analysis of variance was used to assess differences among groups. *P < 0.05, **P < 0.01 vs. HIB. PRE, pre-hibernation group; HIB, hibernation group; IBA, interbout arousal group; POST, post-hibernation group.

Western blot analysis showed significantly decreased (37.39%) HSC70 protein expression in the HIB group compared with the PRE group (*P* < 0.01). Intriguingly, compared with the HIB group, HSC70 protein expression was increased markedly in the IBA (51.39%) and POST (41.67%) groups (both *P* < 0.01). No significant difference was found among the PRE, IBA, and POST groups (Figures 8A,C). The protein expression level of PK did not differ among the four groups (Figures 8A,E).

Conversely, lectin/glyco-antibody microarray analysis revealed significantly decreased (by 30.08%) expression of the terminal SAα2-3Gal structure of glycans on HSC70 in the HIB group compared with the PRE group (*P* < 0.01). This expression increased significantly by 37.66% (*P* < 0.05) and 54.40% (*P* < 0.01) in the IBA and POST groups, respectively. No significant difference was observed among the PRE, IBA, and POST groups (Figures 8B,D). Compared with the PRE group, expression of the terminal SAα2-3Gal structure of PK was significantly reduced (29.40%) in the HIB group (*P* < 0.01). However, the same modification was significantly increased in the IBA (30.91%) and POST (34.41%) groups compared with the HIB group (both *P* < 0.05). No significant difference was detected among the PRE, IBA, and POST groups (Figures 8B,F).

The western blot and lectin/glyco-antibody microarray analyses confirmed that HSC70 and PK were both glycoproteins containing SAα2-3Gal oligosaccharide chains, thus validating the MS results.

## Discussion

In the present study, we observed no change in SOL muscle size and morphology, including muscle wet weight, muscle/body mass ratio, and fiber CSA and distribution, in Daurian ground squirrels during hibernation. These results indicate that the SOL muscle did not atrophy during hibernation. Moreover, we found an oscillatory pattern in the SAα2-3Gal-carrying glycoprotein sialylation level in the SOL muscle of Daurian ground squirrels during hibernation, suggesting its potential involvement in different signaling pathways that could confer protection against disuse muscle atrophy arising from prolonged inactivity. Based on MS identification and bioinformatics analysis, we identified the glycoproteins with terminal SAα2-3Gal modifications. Gene ontology (GO) functional annotation revealed that most glycoproteins with terminal SAα2-3Gal chains are intracellular and macromolecular proteins distributed in organelles and membrane structures, and are primarily binding proteins associated with catalytic and structural functions. Furthermore, GO biological process annotation suggests the potential involvement of these identified glycoproteins in cellular processes, stimulation responses, metabolic processes, and biological regulation. Given the known importance of sialylation in maintaining normal fiber structure and regulating the contractile properties of skeletal muscles and neuromuscular junctions (McDearmon et al., 2003; Johnson et al., 2004; Nilsson et al., 2010; Schwetz et al., 2011), as well as its implications in aging (Marini et al., 2014) and inherited atrophy-related muscular disorders (Tajima et al., 2005; Iwata et al., 2013), the present study supports an association between sialylation during hibernation and protection against disuse atrophy.

Recent studies have suggested that periodic arousal in hibernators compensates for the negative effects of disuse on skeletal muscles during hibernation to prevent disuse muscle atrophy (Lee et al., 2010; Bodine, 2013b). Consistent with these results, our study suggests that the compensatory increase in Sia2-3Gal during interbout arousal could prevent skeletal muscle atrophy in hibernating Daurian ground squirrels. We observed that PTL-I and GSL-I expression levels were significantly increased during periodic interbout arousal, but showed different alteration trends during and after hibernation. Of note, these two lectins are *O*-glycan binders (Wu et al., 1996; Kulkarni et al., 2005; Pan et al., 2005; Tateno et al., 2007), suggesting that the *O*-glycan profiles of glycoproteins in the SOL muscles of Daurian ground squirrels, particularly the *O*-GalNAc–initiated glycan structures, are altered during hibernation. Unlike *N*-glycan structures, which tend to be larger and more branched, *O*-glycans are typically several residues long and are linked to proteins via the hydroxy group of hydroxy amino acids (Spiro, 2002; Willer et al., 2003). *O*-glycosylation has been shown to modulate the gating of Kv channels in skeletal muscle thereby modulating muscle excitability (Schwetz et al., 2011), and play a role in regulating skeletal muscle metabolism and contractile processes, and muscle protein homeostasis (Cieniewski-Bernard et al., 2004, 2006). Taken together, we speculate that increased *O*-glycosylation of proteins in the skeletal muscles of Daurian ground squirrels during interbout arousal may contribute to the innate protective mechanism against disuse muscle atrophy in hibernators.

Utilizing lectin microarray, we found that the GlcNAc and biantennary *N*-glycans in the SOL muscles of Daurian ground squirrels remained unchanged. However, expression of the tri- and tetra-antennary *N*-glycans was significantly increased during hibernation and interbout arousal, and just after final arousal. Lectin histochemistry revealed that PHA-E + L–binding tri- and tetra-antennary complex-type *N*-glycans were moderately bound in the sarcolemma of the SOL muscle in pre-hibernating squirrels, but that binding was significantly elevated in the sarcolemma of hibernating squirrels. Taken together, we showed that the expression levels of tri- and tetra-antennary *N*-glycans of sarcolemma-associated proteins in the skeletal muscles of Daurian ground squirrels were upregulated during hibernation.

Beta-galactoside alpha-2 and 3-sialyltransferases (ST3Gal1–6) transfer sialic acids through α2, 3 linkage to the galactose residues on *N*- and *O*-glycans (Suzuki et al., 2011), and sialidases (NEU1–4) catalyze their removal from glycoconjugates (Fanzani et al., 2012; Miyagi and Yamaguchi, 2012). In the present study, significant downregulation of the mRNA levels of ST3Gal1, ST3Gal2, and ST3Gal5 observed during hibernation, followed by the recovery to normal pre-hibernation level during interbout arousal, was consistent with the alterations in Sia2-3Gal expression noted from the lectin microarray. These results suggest that alterations in the Sia2-3Gal structure during hibernation are likely due to changes in the expression of beta-galactoside alpha-2 and 3-sialyltransferases, including ST3Gal1, ST3Gal2, and ST3Gal5.

The expression levels of major HSPs, e.g., αB-crystallin, Hsp90, Hsp70, Hsp27, and Hsp20, decreased in rats following 2 weeks of hindlimb immobilization, while no significant change was detected in HSC70 expression level (Sakurai et al., 2005). Similarly, no change in HSC70 expression level was observed after 2 weeks of hindlimb unloading in heat shock transcription factor 1–knockdown mice (Yasuhara et al., 2011). These results suggest that HSC70 utilizes a conserved and unique mechanism to protect against skeletal muscle damage. In the present study, we noted an oscillatory pattern in HSC70 expression before, during, and after hibernation. Significant decreases in HSC70 protein expression appeared to coincide with the decrease in Tb, but the HSC70 levels recovered to pre-hibernation levels during interbout arousal and after hibernation, alluding to the likely involvement of a protective mechanism modulating HSC70 level. Our findings, together with previous reports on the recovery of protein and gene expression during interbout arousal (van Breukelen and Martin, 2002; Carey et al., 2003; Storey and Storey, 2010), led us to propose the potential importance of restoring HSC70 protein expression to pre-hibernation levels during interbout arousal in the maintenance of normal physiological function of skeletal muscles during hibernation. Furthermore, the correlation between SAα2-3Gal modification and HSC70 expression in SOL muscle of Daurian ground squirrels before and during hibernation suggests the probable role of sialylation in the prevention of disuse muscle atrophy. Decrease in SAα2-3Gal modification may be due to the inhibition of protein translation and important cellular functions, e.g., sialylation modification, to reduce energy consumption during hibernation (van Breukelen and Martin, 2002; Carey et al., 2003).

The activity of the rate-limiting glycolysis enzyme PK in the SOL muscle, which increased significantly after 21 days of hindlimb immobilization in rats (Bigard et al., 1998), may be associated with the change in metabolic type of skeletal muscle fibers from aerobic to anaerobic under conditions of disuse (Boonyarom and Inui, 2006; Bialek et al., 2011). PK expression in the skeletal muscle cells of hibernating Daurian ground squirrels did not change significantly in the current study, confirming that the SOL muscle fiber type remained constant, unlike in non-hibernating animals. We also observed a similar expression pattern for SAα2-3Gal-modified PK in the course of hibernation to that observed for the HSC70 glycoprotein. Once again, these findings point to the importance of compensatory changes in the sialylation profile during interbout arousal for the maintenance of normal skeletal muscle physiology during hibernation. Similarly, given the known anti-apoptotic function of the 14-3-3 epsilon protein (Roy et al., 1991; Zhang et al., 1999; Fu et al., 2000; Brunet et al., 2002; Milton et al., 2006; Nielsen et al., 2008), the observed restoration of its expression to pre-hibernation levels during interbout arousal in the present study suggests its involvement in preserving normal muscle physiology. This is consistent with the observation that apoptosis was not increased during prolonged hibernation (Rouble et al., 2013), and that the increased muscle cell apoptosis observed in non-hibernating animals led to disuse muscle atrophy (Borisov and Carlson, 2000; Siu et al., 2005; Dupont-Versteegden, 2006).

## Conclusion

We observed an oscillatory pattern in protein glycosylation levels in the SOL muscles of Daurian ground squirrels during prolonged periods of hibernation disuse. This unique glycopattern profile indicates the presence of an underlying mechanism that regulates and maintains protein sialylation homeostasis during periodic hibernation, which could potentially protect hibernators against disuse muscle atrophy. Furthermore, the present study suggests the potential contribution of HSC70, PK, and 14-3-3 epsilon proteins in maintaining normal skeletal muscle physiology and preventing skeletal muscle apoptosis in hibernating squirrels.

## Data Availability Statement

All datasets generated during this study are included in this published article and its Supplementary Material, and all materials generated during this study are available upon request.

## Ethics Statement

The animal study was reviewed and approved by the Northwest University Ethics Committee.

## Author Contributions

KD designed and carried out the experiments and drafted the manuscript. H-JY carried out the experiments and performed the data analysis. S-HX, T-RM, and YL carried out the experiments. H-PW participated in the study design. Y-FG and ZL conceived the study, participated in its design and coordination, and helped in drafting the manuscript.

## Conflict of Interest

The authors declare that the research was conducted in the absence of any commercial or financial relationships that could be construed as a potential conflict of interest.

## Footnotes

1 http://acgt.cs.tau.ac.il/expander/

2 https://sg.idtdna.com/Primerquest/Home/Index

3 https://www.uniprot.org/uniprot/?query=Rodentia+&sort=score

4 https://www.ebi.ac.uk/intact/

## Supplementary Material

FIGURE S1 Lectin microarray layout (TIFF, 7550 kb).

FIGURE S2 Melting curve analysis of PCR products of sialyltransferase and sialidase gene-specific primers in the SOL muscles of Daurian ground squirrels. **(A–D)** Melting curve analysis of 3-sialyltransferases 1 (ST3Gal1) **(A)**, ST3Gal2 **(B)**, ST3Gal3 **(C)** and ST3Gal5 **(D)**. **(E–F)** Melting curve analysis of sialidases NEU1 **(E)** and NEU3 **(F)**.

TABLE S1 Sugar-binding specificities of lectins (TIFF, 92 kb).

TABLE S2 Detailed information on glycoproteins with the SAα2-3Gal structure isolated by MAL-II magnetic particle composites in the SOL muscles of Daurian ground squirrels from the PRE and HIB groups (PDF, 205 kb).

TABLE S3 Significantly enriched KEGG pathways of glycoproteins with the SAα2-3Gal structure in the SOL muscles of Daurian ground squirrels in the PRE group (PDF, 75 kb).

TABLE S4 Significantly enriched KEGG pathways of glycoproteins with the SAα2-3Gal structure in the SOL muscles of Daurian ground squirrels in the HIB group (PDF, 72 kb).

## References

[B1] Apweiler R. Hermjakob H. Sharon N. (1999). On the frequency of protein glycosylation, as deduced from analysis of the SWISS-PROT database. *Biochim. Biophys. Acta Gen. Subj.* 1473 4–8. 10.1016/s0304-4165(99)00165-8 10580125

[B2] Bialek P. Morris C. Parkington J. St Andre M. Owens J. Yaworsky P. (2011). Distinct protein degradation profiles are induced by different disuse models of skeletal muscle atrophy. *Physiol. Genomics* 43 1075–1086. 10.1152/physiolgenomics.00247.2010 21791639 PMC3217324

[B3] Bigard A. X. Boehm E. Veksler V. Mateo P. Anflous K. Ventura-Clapier R. (1998). Muscle unloading induces slow to fast transitions in myofibrillar but not mitochondrial properties. Relevance to skeletal muscle abnormalities in heart failure. *J. Mol. Cell. Cardiol.* 30 2391–2401. 10.1006/jmcc.1998.0798 9925374

[B4] Bodine S. C. (2013a). Disuse-induced muscle wasting. *Int. J. Biochem. Cell Biol.* 45 2200–2208. 10.1016/j.biocel.2013.06.011 23800384 PMC3856924

[B5] Bodine S. C. (2013b). Hibernation: the search for treatments to prevent disuse-induced skeletal muscle atrophy. *Exp. Neurol.* 248 129–135. 10.1016/j.expneurol.2013.06.003 23769906

[B6] Bonaldo P. Sandri M. (2013). Cellular and molecular mechanisms of muscle atrophy. *Dis. Model. Mech.* 6 25–39. 10.1242/dmm.010389 23268536 PMC3529336

[B7] Boonyarom O. Inui K. (2006). Atrophy and hypertrophy of skeletal muscles: structural and functional aspects. *Acta Physiol.* 188 77–89. 10.1111/j.1748-1716.2006.01613.x 16948795

[B8] Borisov A. B. Carlson B. M. (2000). Cell death in denervated skeletal muscle is distinct from classical apoptosis. *Anat. Rec.* 258 305–318. 10.1002/(sici)1097-0185(20000301)258:3<305::aid-ar10>3.0.co;2-a 10705351

[B9] Brunet A. Kanai F. Stehn J. Xu J. Sarbassova D. Frangioni J. V. (2002). 14-3-3 transits to the nucleus and participates in dynamic nucleocytoplasmic transport. *J. Cell Biol.* 156 817–828. 10.1083/jcb.200112059 11864996 PMC2173313

[B10] Carey H. V. Andrews M. T. Martin S. L. (2003). Mammalian hibernation: cellular and molecular responses to depressed metabolism and low temperature. *Physiol. Rev.* 83 1153–1181. 10.1152/physrev.00008.2003 14506303

[B11] Cieniewski-Bernard C. Bastide B. Lefebvre T. Lemoine J. Mounier Y. Michalski J. C. (2004). Identification of O-linked N-acetylglucosamine proteins in rat skeletal muscle using two-dimensional gel electrophoresis and mass spectrometry. *Mol. Cell. Proteomics* 3 577–585. 10.1074/mcp.m400024-mcp200 14985449

[B12] Cieniewski-Bernard C. Mounier Y. Michalski J. C. Bastide B. (2006). O-GlcNAc level variations are associated with the development of skeletal muscle atrophy. *J. Appl. Physiol.* 100 1499–1505. 10.1152/japplphysiol.00865.2005 16357072

[B13] Dang K. Feng B. Gao Y. Hu N. (2016a). Muscle protection during hibernation: role of atrogin-1 and MuRF1,and fiber type transition in Daurian ground squirrels. *Can. J. Zool.* 94 619–629. 10.1139/cjz-2015-0242

[B14] Dang K. Li Y. Z. Gong L. C. Xue W. Wang H. P. Goswami N. (2016b). Stable atrogin-1 (Fbxo32) and MuRF1 (Trim63) gene expression is involved in the protective mechanism in soleus muscle of hibernating Daurian ground squirrels (*Spermophilus dauricus*). *Biol. Open* 5 62–71. 10.1242/bio.015776 26740574 PMC4728309

[B15] Degens H. Alway S. E. (2006). Control of muscle size during disuse, disease, and aging. *Int. J. Sports Med.* 27 94–99. 10.1055/s-2005-837571 16475053

[B16] Dupont-Versteegden E. E. (2006). Apoptosis in skeletal muscle and its relevance to atrophy. *World J. Gastroenterol.* 12 7463–7466. 17167834 10.3748/wjg.v12.i46.7463 PMC4087591

[B17] Fanzani A. Zanola A. Faggi F. Papini N. Venerando B. Tettamanti G. (2012). Implications for the mammalian sialidases in the physiopathology of skeletal muscle. *Skelet. Muscle* 2:23. 10.1186/2044-5040-2-23 23114189 PMC3534598

[B18] Fu Q. Iwase S. Kamiya A. Michikami D. Niimi Y. Mano T. (2000). Leg venous compliance in orthostatic intolerance before and after 14-day head-down bed rest. *Environ. Med.* 44 53–55. 11758569

[B19] Gao Y. F. Arfat Y. Wang H. P. Goswami N. (2018). Muscle atrophy induced by mechanical unloading: mechanisms and potential countermeasures. *Front. Physiol.* 9:235. 10.3389/fphys.2018.00235 29615929 PMC5869217

[B20] Gao Y. F. Wang J. Wang H. P. Feng B. Dang K. Wang Q. (2012). Skeletal muscle is protected from disuse atrophy in hibernating Daurian ground squirrels. *Comp. Biochem. Physiol. A Mol. Integr. Physiol.* 161 296–300. 10.1016/j.cbpa.2011.11.009 22133905

[B21] Gotz S. Garcia-Gomez J. M. Terol J. Williams T. D. Nagaraj S. H. Nueda M. J. (2008). High-throughput functional annotation and data mining with the Blast2GO suite. *Nucleic Acids Res.* 36 3420–3435. 10.1093/nar/gkn176 18445632 PMC2425479

[B22] Gustafsson T. Osterlund T. Flanagan J. N. von Walden F. Trappe T. A. Linnehan R. M. (2010). Effects of 3 days unloading on molecular regulators of muscle size in humans. *J. Appl. Physiol.* 109 721–727. 10.1152/japplphysiol.00110.2009 20538844

[B23] Haliloglu G. Topaloglu H. (2004). Glycosylation defects in muscular dystrophies. *Curr. Opin. Neurol.* 17 521–527. 10.1097/00019052-200410000-00002 15367856

[B24] Haltiwanger R. S. (2002). Regulation of signal transduction pathways in development by glycosylation. *Curr. Opin. Struct. Biol.* 12 593–598. 10.1016/s0959-440x(02)00371-8 12464310

[B25] Haltiwanger R. S. Lowe J. B. (2004). Role of glycosylation in development. *Annu. Rev. Biochem.* 73 491–537. 10.1146/annurev.biochem.73.011303.074043 15189151

[B26] Helenius A. Aebi M. (2004). Roles of N-linked glycans in the endoplasmic reticulum. *Annu. Rev. Biochem.* 73 1019–1049. 10.1146/annurev.biochem.73.011303.073752 15189166

[B27] Iwata Y. Suzuki O. Wakabayashi S. (2013). Decreased surface sialic acid content is a sensitive indicator of muscle damage. *Muscle Nerve* 47 372–378. 10.1002/mus.23632 23382102

[B28] Johnson D. Montpetit M. L. Stocker P. J. Bennett E. S. (2004). The sialic acid component of the beta1 subunit modulates voltage-gated sodium channel function. *J. Biol. Chem.* 279 44303–44310. 10.1074/jbc.m408900200 15316006

[B29] Kohl M. Wiese S. Warscheid B. (2011). Cytoscape: software for visualization and analysis of biological networks. *Methods Mol. Biol.* 696 291–303. 10.1007/978-1-60761-987-1_18 21063955

[B30] Kulkarni K. A. Sinha S. Katiyar S. Surolia A. Vijayan M. Suguna K. (2005). Structural basis for the specificity of basic winged bean lectin for the Tn-antigen: a crystallographic, thermodynamic and modelling study. *FEBS Lett.* 579 6775–6780. 10.1016/j.febslet.2005.11.011 16310781

[B31] Lee K. So H. Gwag T. Ju H. Lee J. W. Yamashita M. (2010). Molecular mechanism underlying muscle mass retention in hibernating bats: role of periodic arousal. *J. Cell. Physiol.* 222 313–319. 10.1002/jcp.21952 19847807

[B32] Li C. Simeone D. M. Brenner D. E. Anderson M. A. Shedden K. A. Ruffin M. T. (2009). Pancreatic cancer serum detection using a lectin/glyco-antibody array method. *J. Proteome Res.* 8 483–492. 10.1021/pr8007013 19072160 PMC2637303

[B33] Lohuis T. D. Harlow H. J. Beck T. D. Iaizzo P. A. (2007). Hibernating bears conserve muscle strength and maintain fatigue resistance. *Physiol. Biochem. Zool.* 80 257–269. 10.1086/513190 17390282

[B34] Lu A. Hu X. Wang Y. Shen X. Li X. Zhu A. (2014). iTRAQ analysis of gill proteins from the zebrafish (*Danio rerio*) infected with *Aeromonas hydrophila*. *Fish Shellfish Immunol.* 36 229–239. 10.1016/j.fsi.2013.11.007 24269520

[B35] Lv J. Zhu Y. X. Liu Y. Q. Xue X. (2015). Distinctive pathways characterize *A. actinomycetemcomitans* and *P. gingivalis*. *Mol. Biol. Rep.* 42 441–449. 10.1007/s11033-014-3785-2 25351486

[B36] Malicdan M. C. Noguchi S. Hayashi Y. K. Nishino I. (2008). Muscle weakness correlates with muscle atrophy and precedes the development of inclusion body or rimmed vacuoles in the mouse model of DMRV/hIBM. *Physiol. Genomics* 35 106–115. 10.1152/physiolgenomics.90219.2008 18628337

[B37] Marini M. Ambrosini S. Sarchielli E. Thyrion G. D. Bonaccini L. Vannelli G. B. (2014). Expression of sialic acids in human adult skeletal muscle tissue. *Acta Histochem.* 116 926–935. 10.1016/j.acthis.2014.03.005 24703356

[B38] Martin-Rendon E. Blake D. J. (2003). Protein glycosylation in disease: new insights into the congenital muscular dystrophies. *Trends Pharmacol. Sci.* 24 178–183. 10.1016/s0165-6147(03)00050-6 12707004

[B39] McDearmon E. L. Combs A. C. Ervasti J. M. (2003). Core 1 glycans on alpha-dystroglycan mediate laminin-induced acetylcholine receptor clustering but not laminin binding. *J. Biol. Chem.* 278 44868–44873. 10.1074/jbc.m307026200 12952987

[B40] Milton A. H. Khaire N. Ingram L. O’Donnell A. J. La Thangue N. B. (2006). 14-3-3 proteins integrate E2F activity with the DNA damage response. *EMBO J.* 25 1046–1057. 10.1038/sj.emboj.7600999 16482218 PMC1409719

[B41] Miyagi T. Yamaguchi K. (2012). Mammalian sialidases: physiological and pathological roles in cellular functions. *Glycobiology* 22 880–896. 10.1093/glycob/cws057 22377912

[B42] Nielsen M. D. Luo X. Biteau B. Syverson K. Jasper H. (2008). 14-3-3 Epsilon antagonizes FoxO to control growth, apoptosis and longevity in *Drosophila*. *Aging Cell* 7 688–699. 10.1111/j.1474-9726.2008.00420.x 18665908 PMC3851013

[B43] Nilsson J. Larson G. Grahn A. (2010). Characterization of site-specific O-glycan structures within the mucin-like domain of alpha-dystroglycan from human skeletal muscle. *Glycobiology* 20 1160–1169. 10.1093/glycob/cwq082 20507882

[B44] Ohira Y. Yoshinaga T. Nomura T. Kawano F. Ishihara A. Nonaka I. (2002). Gravitational unloading effects on muscle fiber size, phenotype and myonuclear number. *Adv. Space Res.* 30 777–781. 10.1016/s0273-1177(02)00395-2 12530363

[B45] Pan L. Li Z. Gong Y. Yu M. Yang K. Pang Y. (2005). Characterization of gp41 gene of Spodoptera litura multicapsid nucleopolyhedrovirus. *Virus Res.* 110 73–79. 10.1016/j.virusres.2005.01.008 15845257

[B46] Qin Y. Zhong Y. Dang L. Zhu M. Yu H. Chen W. (2012). Alteration of protein glycosylation in human hepatic stellate cells activated with transforming growth factor-beta1. *J. Proteomics* 75 4114–4123. 10.1016/j.jprot.2012.05.040 22659384

[B47] Ribeiro J. P. Mahal L. K. (2013). Dot by dot: analyzing the glycome using lectin microarrays. *Curr. Opin. Chem. Biol.* 17 827–831. 10.1016/j.cbpa.2013.06.009 23856055 PMC3823826

[B48] Rouble A. N. Hefler J. Mamady H. Storey K. B. Tessier S. N. (2013). Anti-apoptotic signaling as a cytoprotective mechanism in mammalian hibernation. *PeerJ* 1:e29. 10.7717/peerj.29 23638364 PMC3628845

[B49] Roy R. R. Baldwin K. M. Edgerton V. R. (1991). The plasticity of skeletal muscle: effects of neuromuscular activity. *Exerc. Sport Sci. Rev.* 19 269–312. 1936088

[B50] Rudrappa S. S. Wilkinson D. J. Greenhaff P. L. Smith K. Idris I. Atherton P. J. (2016). Human skeletal muscle disuse atrophy: effects on muscle protein synthesis, breakdown, and insulin resistance-a qualitative review. *Front. Physiol.* 7:361. 10.3389/fphys.2016.00361 27610086 PMC4997013

[B51] Sakurai T. Fujita Y. Ohto E. Oguro A. Atomi Y. (2005). The decrease of the cytoskeleton tubulin follows the decrease of the associating molecular chaperone alphaB-crystallin in unloaded soleus muscle atrophy without stretch. *FASEB J.* 19 1199–1201. 10.1096/fj.04-3060fje 15894563

[B52] Schwetz T. A. Norring S. A. Ednie A. R. Bennett E. S. (2011). Sialic acids attached to O-glycans modulate voltage-gated potassium channel gating. *J. Biol. Chem.* 286 4123–4132. 10.1074/jbc.M110.171322 21115483 PMC3039321

[B53] Siu P. M. Pistilli E. E. Alway S. E. (2005). Apoptotic responses to hindlimb suspension in gastrocnemius muscles from young adult and aged rats. *Am. J. Physiol. Regul. Integr. Comp. Physiol.* 289 R1015–R1026. 15919734 10.1152/ajpregu.00198.2005

[B54] Spiro R. G. (2002). Protein glycosylation: nature, distribution, enzymatic formation, and disease implications of glycopeptide bonds. *Glycobiology* 12 43R–56R. 10.1093/glycob/12.4.43r 12042244

[B55] Stalnaker S. H. Aoki K. Lim J. M. Porterfield M. Liu M. Satz J. S. (2011). Glycomic analyses of mouse models of congenital muscular dystrophy. *J. Biol. Chem.* 286 21180–21190. 10.1074/jbc.M110.203281 21460210 PMC3122180

[B56] Storey K. B. Storey J. M. (2010). Metabolic rate depression: the biochemistry of mammalian hibernation. *Adv. Clin. Chem.* 52 77–108. 21275340

[B57] Suzuki O. Kanai T. Nishikawa T. Yamamoto Y. Noguchi A. Takimoto K. (2011). Adult onset cardiac dilatation in a transgenic mouse line with Galbeta1,3GalNAc alpha2,3-sialyltransferase II (ST3Gal-II) transgenes: a new model for dilated cardiomyopathy. *Proc. Jpn. Acad. Ser. B Phys. Biol. Sci.* 87 550–562. 10.2183/pjab.87.550 21986317 PMC3313694

[B58] Tajima Y. Uyama E. Go S. Sato C. Tao N. Kotani M. (2005). Distal myopathy with rimmed vacuoles: impaired O-glycan formation in muscular glycoproteins. *Am. J. Pathol.* 166 1121–1130. 15793292 10.1016/S0002-9440(10)62332-2 PMC1602383

[B59] Tateno H. Uchiyama N. Kuno A. Togayachi A. Sato T. Narimatsu H. (2007). A novel strategy for mammalian cell surface glycome profiling using lectin microarray. *Glycobiology* 17 1138–1146. 10.1093/glycob/cwm084 17693441

[B60] van Breukelen F. Martin S. L. (2002). Reversible depression of transcription during hibernation. *J. Comp. Physiol. B* 172 355–361. 10.1007/s00360-002-0256-1 12122451

[B61] Willer T. Valero M. C. Tanner W. Cruces J. Strahl S. (2003). O-mannosyl glycans: from yeast to novel associations with human disease. *Curr. Opin. Struct. Biol.* 13 621–630. 10.1016/j.sbi.2003.09.003 14568618

[B62] Wu A. M. Wu J. H. Song S. C. Kabat E. A. (1996). Bandeiraea (Griffonia) simplicifolia lectin-I, isolectin A4, reacting with Tn (Ga1NAc alpha1–> Ser/Thr) or galabiose (Ga1 alpha1–> 4Ga1) containing ligands. *FEBS Lett.* 398 183–186. 10.1016/s0014-5793(96)01227-6 8977103

[B63] Wu J. Xie X. Liu Y. He J. Benitez R. Buckanovich R. J. (2012). Identification and confirmation of differentially expressed fucosylated glycoproteins in the serum of ovarian cancer patients using a lectin array and LC-MS/MS. *J. Proteome Res.* 11 4541–4552. 10.1021/pr300330z 22827608

[B64] Yang G. Chu W. Zhang H. Sun X. Cai T. Dang L. (2013a). Isolation and identification of mannose-binding proteins and estimation of their abundance in sera from hepatocellular carcinoma patients. *Proteomics* 13 878–892. 10.1002/pmic.201200018 23300094

[B65] Yang G. Cui T. Chen Q. Ma T. Li Z. (2012). Isolation and identification of native membrane glycoproteins from living cell by concanavalin A-magnetic particle conjugates. *Anal. Biochem.* 421 339–341. 10.1016/j.ab.2011.10.033 22079135

[B66] Yang G. Cui T. Wang Y. Sun S. Ma T. Wang T. (2013b). Selective isolation and analysis of glycoprotein fractions and their glycomes from hepatocellular carcinoma sera. *Proteomics* 13 1481–1498. 10.1002/pmic.201200259 23436760

[B67] Yasuhara K. Ohno Y. Kojima A. Uehara K. Beppu M. Sugiura T. (2011). Absence of heat shock transcription factor 1 retards the regrowth of atrophied soleus muscle in mice. *J. Appl. Physiol.* 111 1142–1149. 10.1152/japplphysiol.00471.2011 21817109

[B68] Yu H. Zhu M. Qin Y. Zhong Y. Yan H. Wang Q. (2012). Analysis of glycan-related genes expression and glycan profiles in mice with liver fibrosis. *J. Proteome Res.* 11 5277–5285. 10.1021/pr300484j 23043565

[B69] Zhang L. Chen J. Fu H. (1999). Suppression of apoptosis signal-regulating kinase 1-induced cell death by 14-3-3 proteins. *Proc. Natl. Acad. Sci. U.S.A.* 96 8511–8515. 10.1073/pnas.96.15.8511 10411906 PMC17547

[B70] Zhang Z. G. Niu X. Y. Lu A. P. Xiao G. G. (2015). Effect of curcumin on aged *Drosophila melanogaster*: a pathway prediction analysis. *Chin. J. Integr. Med.* 21 115–122. 10.1007/s11655-013-1333-2 24155070

