# Supplementary figures and images for: Remarkable Homeostasis of Protein Sialylation in Skeletal Muscles of Hibernating Daurian Ground Squirrels (Spermophilus dauricus)
Source: Front Physiol. 2020 Feb 7;11:37. doi: 10.3389/fphys.2020.00037 (PMC7020753; doi:10.3389/fphys.2020.00037)

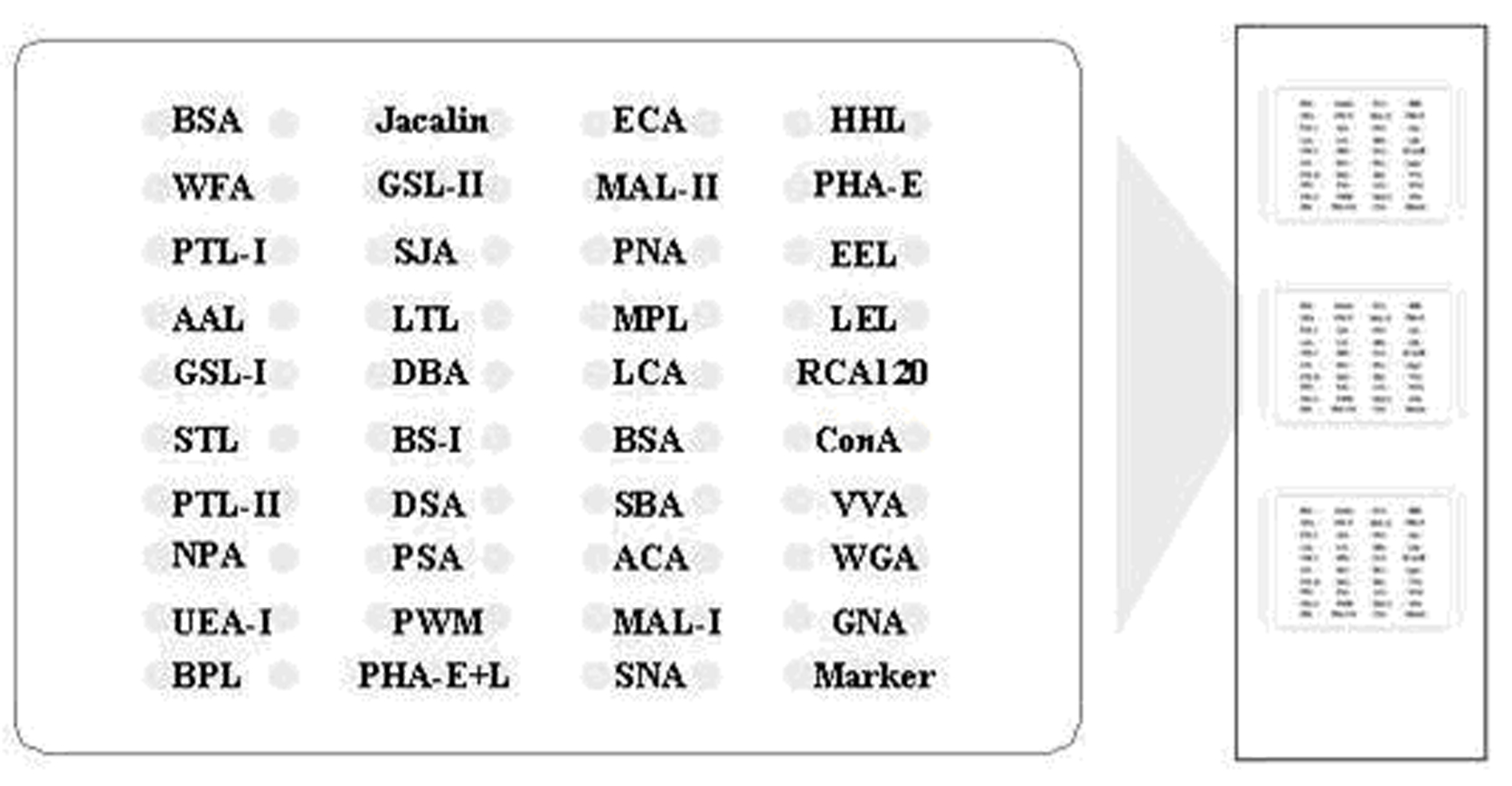

Supplement: FIGURE S1 — Lectin microarray layout (TIFF, 7550 kb). [file Image_1.TIF]

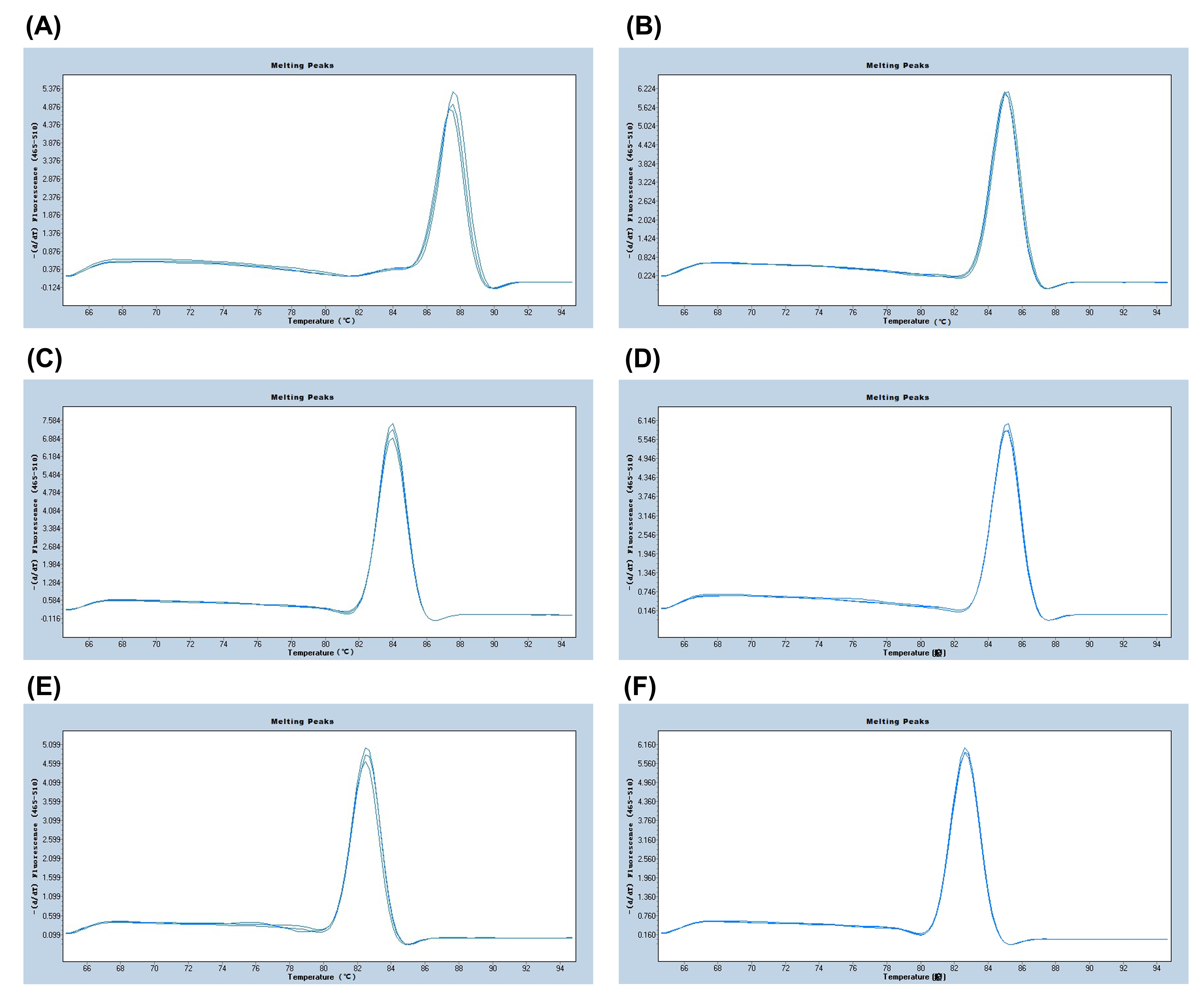

Supplement: FIGURE S2 — Melting curve analysis of PCR products of sialyltransferase and sialidase gene-specific primers in the SOL muscles of Daurian ground squirrels. (A–D) Melting curve analysis of 3-sialyltransferases 1 (ST3Gal1) (A), ST3Gal2 (B), ST3Gal3 (C) and ST3Gal5 (D). (E–F) Melting curve analysis of sialidases NEU1 (E) and NEU3 (F). [file Image_2.TIF]
