# Supplementary material for: Remarkable Homeostasis of Protein Sialylation in Skeletal Muscles of Hibernating Daurian Ground Squirrels (Spermophilus dauricus)
Source: Front Physiol. 2020 Feb 7;11:37. doi: 10.3389/fphys.2020.00037 (PMC7020753; doi:10.3389/fphys.2020.00037)
Supplement: TABLE S1 — Sugar-binding specificities of lectins (TIFF, 92 kb). [file Table_1.DOCX]

**Table S1. Sugar-binding specificities of lectins.**

| **Lectin name** | **Abbreviation** | **Specificity** |
| --- | --- | --- |
| Erythrina cristagalli | ECA | Galβ-1,4GlcNAc |
| Artocapus integrifolia | Jacalin | Galβ1-3GalNAcα-Ser/Thr（T）and GalNAcα- Ser/Thr（T） |
| Hippeastrum Hybrid Lectin | HHL | Non-substitutedα-1,6Man |
| Wisteria Floribunda Lectin | WFA | Terminal GalNAc |
| Griffonia (Bandeiraea) Simplicifolia Lectin II | GSL-II | GlcNAc and galactosylated *N*-glycans |
| [Maackia Amurensis Lectin II](http://www.vectorlabs.com/products.asp?catID=231&locID=168) | MAL-II | SAα2-3Gal |
| [Sophora Japonica Agglutinin](http://www.vectorlabs.com/products.asp?catID=248&locID=168) | SJA | terminal in GalNAc and Gal |
| Phaseolus vulgaris Agglutinin (E) | PHA-E | Bisecting GlcNAc and biantennary *N*-glycans |
| [Psophocarpus Tetragonolobus Lectin I](http://www.vectorlabs.com/products.asp?catID=240&locID=168) | PTL-I | αGalNAc and Gal |
| [Peanut Agglutinin](http://www.vectorlabs.com/products.asp?catID=235&locID=168) | PNA | Galβ1-3GalNAcα-Ser/Thr（T） |
| [Aleuria Aurantia Lectin](http://www.vectorlabs.com/products.asp?catID=207&locID=168) | AAL | Fucose |
| [Euonymus Europaeus Lectin](http://www.vectorlabs.com/products.asp?catID=218&locID=168) | EEL | Galα1-3（Fucα1-2）Gal |
| [Lotus Tetragonolobus Lectin](http://www.vectorlabs.com/products.asp?catID=228&locID=168) | LTL | Fucoseα-1,3GlcNAc（core fucose），SA-Lex and Lex |
| [Maclura Pomifera Lectin](http://www.vectorlabs.com/products.asp?catID=232&locID=168) | MPL | αGalNAc |
| Dolichos Biflorus Agglutinin | DBA | GalNAcα-Ser/Thr（Tn） and GalNAcα1-3Gal |
| [Lycopersicon Esculentum Lectin](http://www.vectorlabs.com/products.asp?catID=229&locID=168) | LEL | Poly-LacNAc and （GlcNAc）n |
| [Griffonia (Bandeiraea) Simplicifolia Lectin I](http://www.vectorlabs.com/products.asp?catID=222&locID=168) | GSL-I | αGalNAc,GalNAcα-Ser/Thr（Tn）and αGal |
| [Lens Culinaris Agglutinin](http://www.vectorlabs.com/products.asp?catID=227&locID=168) | LCA | Fucoseα-1,6GlcNAc（core fucose） |
| [Ricinus Communis Agglutinin I](http://www.vectorlabs.com/products.asp?catID=242&locID=168) | RCA120 | β-gal |
| Bandeiraea simplicifolia | BS-I | α-gal and α-GalNAc |
| Solanum Tuberosum (Potato) Lectin | STL | Oligomers of GlcNAc |
| Canavalia ensiformis | ConA | branched and terminal mannose,terminal GlcNAc |
| [Psophocarpus Tetragonolobus Lectin II](http://www.vectorlabs.com/products.asp?catID=241&locID=168) | PTL-II | Gal |
| Datura stramonium | DSA | GlcNAc |
| [Soybean Agglutinin](http://www.vectorlabs.com/products.asp?catID=249&locID=168) | SBA | Terminal GalNAc（especially GalNAcα1-3Gal） |
| Vicia Villosa Lectin | VVA | GalNAc and GalNAcα-Ser/Thr（Tn） |
| Narcissus Pseudonarcissus Lectin | NPL | Non-substitutedα-1,6Man |
| Pisum Sativum Agglutinin | PSA | Fucoseα-1,6GlcNAc（core fucose） |
| Amaranthus caudatus | ACA | Galβ1-3GalNAcα-Ser/Thr（T-antigen） |
| Triticum vulgaris | WGA | Mμltivalent SA and （GlcNAc）n |
| Ulex Europaeus Agglutinin I | UEA-I | Fucoseα1-2Galβ1-4Glc（NAc） |
| Phytolacca americana | PWM | GlcNAc |
| [Maackia Amurensis Lectin I](http://www.vectorlabs.com/products.asp?catID=230&locID=168) | MAL-I | Galβ-1,4GlcNAc |
| Galanthus nivalis | GNA | Terminalα-1,3 mannose |
| [Bauhinia Purpurea Lectin](http://www.vectorlabs.com/products.asp?catID=210&locID=168) | BPL | Galβ1-3GalNAc |
| Phaseolus vulgaris Agglutinin（E+L） | PHA-E+L | Bisecting GlcNAc and biantennary *N*-glycans and tetraantennary complex-type *N*-glycan |
| Sambucus Nigra Lectin | SNA | SAα2-6Gal |
