# Supplementary material for: Remarkable Homeostasis of Protein Sialylation in Skeletal Muscles of Hibernating Daurian Ground Squirrels (Spermophilus dauricus)
Source: Front Physiol. 2020 Feb 7;11:37. doi: 10.3389/fphys.2020.00037 (PMC7020753; doi:10.3389/fphys.2020.00037)
Supplement: TABLE S2 — Detailed information on glycoproteins with the SAα2-3Gal structure isolated by MAL-II magnetic particle composites in the SOL muscles of Daurian ground squirrels from the PRE and HIB groups (PDF, 205 kb). [file Table_2.DOC]

**Table S2. Detailed information on glycoproteins with the SAα2-3Gal structure isolated by MAL-II magnetic particle composites in the SOL muscle of Daurian ground squirrels from the PRE and HIB groups.**

| Number | Accession number | Protein name | P/H | Cover  Percent | MW | PI |
| --- | --- | --- | --- | --- | --- | --- |
| 1 | B2RWX0 | Myosin, heavy polypeptide 1, skeletal muscle, adult | P, H | 12.10% | 223344.79 | 5.61 |
| 2 | G3UW82 | MCG140437, isoform CRA_d | P, H | 11.02% | 223216.86 | 5.61 |
| 3 | F1M8F6 | Uncharacterized protein | P, H | 8.85% | 223692.75 | 6.04 |
| 4 | Q5SX39 | Myosin-4 | P | 9.64% | 222856.23 | 5.58 |
| 5 | P10719 | ATP synthase subunit beta, mitochondrial | P, H | 31.38% | 56352.87 | 5.18 |
| 6 | P13539 | Myosin-6 OS=Mesocricetus auratus | P | 7.84% | 223624.68 | 5.54 |
| 7 | P68136 | Actin, alpha skeletal muscle | P | 35.54% | 42050.59 | 5.23 |
| 8 | G5BL81 | ATP synthase subunit alpha | P, H | 21.88% | 59784.03 | 9.17 |
| 9 | Q3UGS0 | Putative uncharacterized protein | P, H | 27.73% | 41764.35 | 5.38 |
| 10 | B1WBU9 | Alpha-1,4 glucan phosphorylase | P | 11.76% | 97321.21 | 6.65 |
| 11 | I3LZY6 | Uncharacterized protein | H | 14.74% | 57357.85 | 5.06 |
| 12 | Q9CXK3 | Putative uncharacterized protein | H | 20.54% | 41946.46 | 5.3 |
| 13 | I3MA46 | Uncharacterized protein | P | 11.45% | 62029.26 | 7.62 |
| 14 | I3MIZ9 | Uncharacterized protein | P, H | 20.04% | 53361.99 | 5.21 |
| 15 | H0VAR5 | Uncharacterized protein | P, H | 9.09% | 59726.84 | 5.54 |
| 16 | G5B5H9 | Myosin-13 | P, H | 3.70% | 224120.68 | 5.74 |
| 17 | I3M806 | Uncharacterized protein | P | 10.65% | 59475.96 | 5.32 |
| 18 | I3MB56 | Pyruvate kinase | P, H | 15.44% | 57910.36 | 6.88 |
| 19 | P63017 | Heat shock cognate 70 kDa protein | P, H | 12.07% | 70870.22 | 5.37 |
| 20 | E0ZS30 | Beta-geo | P | 5.40% | 146247.74 | 5.07 |
| 21 | I3MKZ0 | Uncharacterized protein | P, H | 14.05% | 52781.06 | 5.89 |
| 22 | I3LYW1 | Uncharacterized protein | P, H | 10.11% | 51251.92 | 9.49 |
| 23 | I3MRL5 | Uncharacterized protein | P, H | 4.65% | 163966.27 | 5.79 |
| 24 | A0A091CNE8 | Cytochrome b-c1 complex subunit 1, mitochondrial | P, H | 9.17% | 52632.03 | 5.98 |
| 25 | I3MF96 | Uncharacterized protein | P | 9.17% | 52685.99 | 5.86 |
| 26 | I3ND33 | Uncharacterized protein | P | 9.80% | 71079.24 | 5.71 |
| 27 | P16627 | Heat shock 70 kDa protein 1-like | P, H | 9.83% | 70636.44 | 5.91 |
| 28 | G5ARA1 | Lamin-A/C | P | 6.99% | 87190.64 | 8.05 |
| 29 | Q9R0H4 | Beta cardiac myosin heavy chain | P, H | 26.99% | 19117.25 | 4.84 |
| 30 | I3N486 | Uncharacterized protein | P, H | 24.26% | 22500.89 | 6.09 |
| 31 | P23928 | Alpha-crystallin B chain | P, H | 25.14% | 20088.58 | 6.76 |
| 32 | O35129 | Prohibitin-2 | P, H | 14.72% | 33295.97 | 9.83 |
| 33 | I3MMU3 | Uncharacterized protein | P, H | 9.58% | 44631.14 | 4.72 |
| 34 | I3M611 | Uncharacterized protein | P | 6.39% | 85604.36 | 6.1 |
| 35 | I3MF52 | Uncharacterized protein | P, H | 8.53% | 79407.4 | 5.71 |
| 36 | I3MMW4 | Uncharacterized protein | P | 9.01% | 48165.22 | 5.04 |
| 37 | G5BV89 | Cytochrome b-c1 complex subunit 2, mitochondrial | P, H | 8.17% | 48324.24 | 8.93 |
| 38 | Q9EQP5 | Prolargin | P, H | 12.20% | 43178.87 | 9.51 |
| 39 | I3N4U1 | Uncharacterized protein | P, H | 11.60% | 43116.16 | 6.05 |
| 40 | Q3TKF8 | Putative uncharacterized protein | H | 7.48% | 72349.1 | 5.09 |
| 41 | I3M8L3 | Calcium-transporting ATPase | P, H | 3.69% | 110579.55 | 5.15 |
| 42 | G5BRD7 | endoplasmic reticulum calcium ATPase 1 | P, H | 3.31% | 112966.62 | 5.39 |
| 43 | P50609 | Fibromodulin | P, H | 8.24% | 43218.42 | 5.67 |
| 44 | G3HWE9 | Tubulin alpha-4A chain | P, H | 8.78% | 48328.02 | 4.9 |
| 45 | P86217 | NADH dehydrogenase [ubiquinone] iron-sulfur protein 3, mitochondrial | P, H | 39.36% | 11106.52 | 5.64 |
| 46 | P48670 | Vimentin | P, H | 9.15% | 51848.06 | 4.94 |
| 47 | Q3TIP0 | Putative uncharacterized protein | P, H | 5.29% | 100217.57 | 5.08 |
| 48 | Q8VHX6 | Filamin-C | H | 1.65% | 291115.82 | 5.63 |
| 49 | G5CAX4 | Heat shock cognate 71 kDa protein | P, H | 8.45% | 57599.21 | 5.66 |
| 50 | I3M6D6 | Myoglobin | P | 24.68% | 17190.59 | 7.94 |
| 51 | I3M7H9 | Uncharacterized protein | P, H | 12.97% | 25830.74 | 7.82 |
| 52 | I3NB07 | Uncharacterized protein | P | 22.92% | 16771.3 | 8.92 |
| 53 | I3LWZ7 | Uncharacterized protein | P | 8.12% | 39328.08 | 4.74 |
| 54 | I3M0Z1 | Uncharacterized protein | P | 1.85% | 250727.87 | 7.71 |
| 55 | A0A091D3B5 | Tropomyosin alpha-1 chain | P, H | 5.63% | 32774.28 | 4.69 |
| 56 | P58775 | Tropomyosin beta chain | H | 5.63% | 32836.34 | 4.66 |
| 57 | G3V908 | Protein Kb15 | P, H | 4.32% | 57608.89 | 8.04 |
| 58 | I3MVD0 | Uncharacterized protein | P | 4.95% | 51451.19 | 5.68 |
| 59 | H0VE97 | Pyruvate dehydrogenase E1 component subunit alpha | P, H | 5.64% | 43391.33 | 8.61 |
| 60 | I3N008 | Uncharacterized protein | P, H | 17.57% | 16048.13 | 6.16 |
| 61 | A0A061ILH1 | Anionic trypsin-2-like protein | P, H | 8.16% | 25833.25 | 6.85 |
| 62 | G3HL18 | Anionic trypsin-2 | P, H | 7.94% | 26732.19 | 7.45 |
| 63 | Q99KI0 | Aconitate hydratase, mitochondrial | P, H | 3.85% | 85462.49 | 8.08 |
| 64 | H0VU95 | Uncharacterized protein | P, H | 28.36% | 14424.39 | 7.82 |
| 65 | I3MBI5 | Uncharacterized protein | P, H | 5.87% | 66909.9 | 9.19 |
| 66 | P86210 | Alpha-enolase | P, H | 12.56% | 23836.89 | 5.32 |
| 67 | H0V0E4 | Uncharacterized protein | P, H | 5.13% | 49902.78 | 5.32 |
| 68 | I3ND93 | Uncharacterized protein | H | 5.18% | 49670.28 | 4.78 |
| 69 | A0A091D2L5 | Pyruvate dehydrogenase E1 component subunit beta, mitochondrial | H | 7.80% | 40124.85 | 5.4 |
| 70 | P02468 | Laminin subunit gamma-1 | P, H | 1.24% | 177296.82 | 5.08 |
| 71 | P97617 | Glyceraldehyde 3-phosphate dehydrogenase | P, H | 12.05% | 9129.44 | 9.25 |
| 72 | Q7TQ48 | Sarcalumenin | P, H | 3.19% | 99182.8 | 4.39 |
| 73 | D3ZCI9 | Uncharacterized protein | P | 15.76% | 18946.27 | 5.02 |
| 74 | H0VLU0 | Uncharacterized protein | P | 13.61% | 21465.21 | 5.02 |
| 75 | P68361 | Tubulin alpha-1B chain | P | 6.43% | 50151.07 | 4.94 |
| 76 | Q0QF17 | Succinate dehydrogenase [ubiquinone] flavoprotein subunit, mitochondrial | P, H | 4.72% | 59933.07 | 5.83 |
| 77 | H0W278 | Uncharacterized protein | P, H | 3.41% | 54616.16 | 7.36 |
| 78 | H0W6N6 | Uncharacterized protein | P | 3.39% | 54462.78 | 6.81 |
| 79 | I3MBW8 | Uncharacterized protein | P, H | 3.00% | 74846.67 | 8.03 |
| 80 | I3MHI9 | Uncharacterized protein | P, H | 7.35% | 43052.53 | 6.61 |
| 81 | I3MIA3 | Calcium-transporting ATPase | P | 2.21% | 114553.51 | 5.2 |
| 82 | I3MQ98 | Uncharacterized protein | P, H | 1.90% | 155041.79 | 5.18 |
| 83 | A0A061IB69 | Fructose-bisphosphate aldolase | P | 5.26% | 45280.06 | 8.48 |
| 84 | I3MA99 | Uncharacterized protein | H | 3.96% | 72286.21 | 8.28 |
| 85 | H0V4M6 | Uncharacterized protein | H | 1.93% | 94691.46 | 6.01 |
| 86 | A0A091DRY0 | Uncharacterized protein | P | 2.95% | 49520.67 | 9.08 |
| 87 | D7PGV9 | Apolipoprotein A-I preproprotein | P | 8.33% | 30502.1 | 5.7 |
| 88 | G3IBG3 | Ubiquitin activating enzyme E1 | P | 2.55% | 117696.41 | 5.42 |
| 89 | H0V894 | Uncharacterized protein | P, H | 3.88% | 50675.31 | 8.63 |
| 90 | H0VNJ9 | Uncharacterized protein | P | 3.26% | 56607.93 | 6.63 |
| 91 | I3LXM9 | Uncharacterized protein | P | 2.23% | 161710.49 | 5.57 |
| 92 | I3M435 | Uncharacterized protein | P | 7.03% | 45359.31 | 9.14 |
| 93 | I3M5P3 | Uncharacterized protein | P | 4.76% | 54441.94 | 5.34 |
| 94 | I3ML01 | Uncharacterized protein | P, H | 11.07% | 26905.27 | 7.85 |
| 95 | P11598 | Protein disulfide-isomerase A3 | P | 4.36% | 56622.69 | 5.88 |
| 96 | P68369 | Tubulin alpha-1A chain | P | 6.43% | 50135.07 | 4.94 |
| 97 | Q5FW75 | Actinin alpha 2 | P | 2.91% | 103917.52 | 5.31 |
| 98 | Q958H5 | Cytochrome c oxidase subunit 2 | P | 7.49% | 25807.71 | 4.68 |
| 99 | P14408 | Fumarate hydratase, mitochondrial | P, H | 6.51% | 54463.27 | 9.06 |
| 100 | G5APM7 | Alpha-actinin-2 | P | 2.91% | 103838.45 | 5.31 |
| 101 | H0UTX5 | Uncharacterized protein | P, H | 7.72% | 29803.71 | 5.57 |
| 102 | Q6AY07 | Fructose-bisphosphate aldolase | P | 6.04% | 39491.5 | 7.07 |
| 103 | A0A091E0X0 | Myozenin-1 | P, H | 4.45% | 31073.67 | 9.03 |
| 104 | A0A091DSG0 | Sickle tail protein like protein | P, H | 0.37% | 205053.5 | 7.98 |
| 105 | A0A091E3C8 | Uncharacterized protein | P, H | 4.94% | 18961.47 | 9.67 |
| 106 | F7CE80 | Protein RIC-3 （Fragment） | P, H | 36.36% | 2726.34 | 8.5 |
| 107 | G3HWJ1 | Poly [ADP-ribose] polymerase 10 （Fragment） | P, H | 0.91% | 107667.54 | 4.77 |
| 108 | Q3TLV7 | Putative uncharacterized protein | P | 0.94% | 103763.89 | 4.92 |
| 109 | G5APW8 | Neurofilament heavy polypeptide | P, H | 0.91% | 108739.67 | 6.29 |
| 110 | H0W8Q3 | Uncharacterized protein | H | 1.32% | 83644.86 | 6.45 |
| 111 | G5CAX9 | Myomesin-1 | P, H | 0.83% | 187076.1 | 5.97 |
| 112 | Q5PQS3 | Ventricular zone-expressed PH domain-containing protein homolog 1 | P, H | 0.84% | 94461.87 | 6.56 |
| 113 | H0VR41 | Uncharacterized protein | H | 1.14% | 57087.86 | 5.93 |
| 114 | I3M5D1 | Uncharacterized protein | P, H | 1.61% | 71375.7 | 5.67 |
| 115 | I3MCN4 | Uncharacterized protein | P, H | 1.84% | 73852.21 | 9.85 |
| 116 | P35969 | Vascular endothelial growth factor receptor 1 | P | 1.05% | 149874.44 | 8.62 |
| 117 | Q14A95 | MCG9666, isoform CRA_d | P, H | 3.77% | 30147.69 | 9.37 |
| 118 | Q9D7Y7 | Putative uncharacterized protein | P, H | 4.86% | 26406.64 | 8.22 |
| 119 | O08709 | Peroxiredoxin-6 | P | 3.13% | 24870.34 | 5.71 |
| 120 | A0A061IJN4 | Retroviral matrix containing protein | P, H | 1.93% | 46531.53 | 5.15 |
| 121 | D4A582 | Protein Zdbf2 | P, H | 0.36% | 245394.46 | 4.81 |
| 122 | Q2Q7P0 | ENH1 | P | 1.86% | 63299.54 | 8.61 |
| 123 | E9QNY8 | Sacsin | H | 0.17% | 520685.48 | 6.64 |
| 124 | G3I6E7 | Protransforming growth factor alpha | P, H | 5.21% | 22551.92 | 8.6 |
| 125 | G3I6Z0 | snRNA-activating protein complex subunit 4 | P, H | 0.61% | 146290.53 | 8.67 |
| 126 | G5AS67 | Arf-GAP with Rho-GAP domain, ANK repeat and PH domain-containing protein 1 | P, H | 0.56% | 158732.75 | 5.87 |
| 127 | Q07417 | Short-chain specific acyl-CoA dehydrogenase, mitochondrial | P, H | 3.16% | 44889.1 | 8.68 |
| 128 | A0A091CPY5 | UTP--glucose-1-phosphate uridylyltransferase | P, H | 2.62% | 55768.19 | 7.69 |
| 129 | G5CA63 | Putative G-protein coupled receptor 112 | P | 0.33% | 329187.87 | 6.3 |
| 130 | H0UYP2 | Uncharacterized protein | P, H | 1.57% | 63343.19 | 8.27 |
| 131 | H0V0I2 | Uncharacterized protein | P, H | 1.21% | 82287.12 | 5.21 |
| 132 | H0V534 | Uncharacterized protein | P, H | 5.21% | 21345.2 | 8.46 |
| 133 | H0VB93 | Uncharacterized protein | P, H | 0.22% | 528923 | 5.7 |
| 134 | H0VLC2 | NADH dehydrogenase [ubiquinone] 1 subunit C2 | H | 9.24% | 14059.19 | 7.88 |
| 135 | I3M1Z3 | Cytochrome c oxidase subunit 6A, mitochondrial | P, H | 12.50% | 10707.1 | 9.3 |
| 136 | I3M827 | Sulfurtransferase | H | 4.38% | 32954.82 | 5.74 |
| 137 | A0A091CSU2 | Isocitrate dehydrogenase [NAD] subunit, mitochondrial | P | 4.20% | 41944.87 | 7.21 |
| 138 | P02600 | Myosin light chain 1/3, skeletal muscle isoform | H | 5.82% | 20679.39 | 4.99 |
| 139 | I3N5J9 | Uncharacterized protein | P, H | 3.10% | 40205.39 | 6.53 |
| 140 | I3NG08 | Uncharacterized protein | P, H | 2.70% | 38571.37 | 8.78 |
| 141 | O08756 | 3-hydroxyacyl-CoA dehydrogenase type-2 | P, H | 4.60% | 27418.3 | 8.53 |
| 142 | O88806 | Protein-arginine deiminase type-1 | P | 1.66% | 73856.53 | 6.71 |
| 143 | P14668 | Annexin A5 | P, H | 2.82% | 35744.09 | 4.92 |
| 144 | P86225 | Isocitrate dehydrogenase [NAD] subunit alpha, mitochondrial | P, H | 7.46% | 14881.28 | 9.15 |
| 145 | Q2KML1 | Chromodomain helicase DNA-binding protein 3 short isoform | P | 0.52% | 218031.69 | 7.11 |
| 146 | I3MPV9 | Uncharacterized protein | P | 3.75% | 28483 | 8.84 |
| 147 | G5B2W9 | ADP/ATP translocase 1 | H | 3.18% | 31734.64 | 9.78 |
| 148 | Q9WVK7 | Hydroxyacyl-coenzyme A dehydrogenase, mitochondrial | H | 3.50% | 34447.41 | 8.83 |
| 149 | Q5SWW4 | Mediator of RNA polymerase II transcription subunit 13 | P | 0.55% | 238588.43 | 5.42 |
| 150 | G3HWW6 | Pre-mRNA-splicing factor SYF1 | P | 0.94% | 100007.45 | 5.87 |
| 151 | B2RZD6 | Ndufa4 protein | P, H | 14.63% | 9326.67 | 9.52 |
| 152 | A0A061I8C8 | Elongation factor Tu | P | 3.88% | 44955.46 | 6.88 |
| 153 | A0A061IDE1 | Myotubularin-related protein 10 | H | 1.16% | 78944.65 | 8.86 |
| 154 | A0A061IIN7 | Myotubularin-related protein 11 | P | 1.57% | 78454.36 | 7.88 |
| 155 | A0A061IKJ9 | Fibronectin type III domain-containing protein 7 | H | 1.16% | 72737.82 | 8.38 |
| 156 | A0A061ILK1 | Coiled-coil domain-containing protein 85C-like protein | H | 9.82% | 12426.76 | 5.43 |
| 157 | A0A087WNR9 | Ligand-dependent nuclear receptor corepressor-like protein | P | 18.37% | 5340.2 | 11.63 |
| 158 | A0A091CLK9 | Complement receptor type 2 | H | 0.51% | 194637.92 | 6.16 |
| 159 | A0A091CQU6 | Obscurin | P | 0.10% | 915869.23 | 5.43 |
| 160 | A0A091CS42 | Serine/threonine-protein kinase SMG1 | H | 0.19% | 409049.37 | 5.96 |
| 161 | Q9CR62 | Mitochondrial 2-oxoglutarate/malate carrier protein | H | 3.18% | 34154.51 | 9.95 |
| 162 | A0A091CY14 | Signal peptide, CUB and EGF-like domain-containing protein 3 | P | 1.05% | 104681.4 | 7.88 |
| 163 | A0A091CZJ7 | Transmembrane protein 214 | P | 2.24% | 70253.12 | 9.03 |
| 164 | A0A091D2D9 | Polyamine-modulated factor 1-binding protein 1 | H | 0.85% | 110243.44 | 6.02 |
| 165 | A0A091D576 | BTB/POZ domain-containing protein KCTD3 | P | 1.29% | 84719.2 | 6.44 |
| 166 | A0A091D5L9 | Zinc finger protein 518B | H | 1.63% | 88172.69 | 9.38 |
| 167 | A0A091D8B1 | Cathepsin F | P | 1.99% | 44908.91 | 6.36 |
| 168 | I3NG05 | Uncharacterized protein | P | 5.58% | 23688.44 | 8.32 |
| 169 | A0A091DBF5 | Myosin-VIIb | P | 0.36% | 252045.8 | 9.03 |
| 170 | A0A091DCS5 | Histone-lysine N-methyltransferase, H3 lysine-79 specific | P | 0.82% | 182250.08 | 9.24 |
| 171 | I3NGX0 | Uncharacterized protein | H | 5.68% | 28166.34 | 4.97 |
| 172 | A0A091DF02 | Transducin-like enhancer protein 6 | H | 1.55% | 56677.72 | 9.11 |
| 173 | A0A091DFA9 | Uncharacterized protein | H | 0.54% | 196803.69 | 5.88 |
| 174 | A0A091DFM8 | Stereocilin | P | 0.35% | 215851.8 | 5.31 |
| 175 | A0A091DFW9 | CASP8-associated protein 2 | H | 0.56% | 223020.77 | 6.46 |
| 176 | A0A091DIJ1 | Transcription elongation factor A protein-like 3 | P | 4.50% | 22518.67 | 5.68 |
| 177 | A0A091DJ30 | Uncharacterized protein | H | 12.24% | 5986.14 | 4.08 |
| 178 | I3N5I3 | Uncharacterized protein | H | 3.13% | 25700.85 | 4.86 |
| 179 | A0A091DJZ6 | HLA class II histocompatibility antigen, DP beta 1 chain | H | 8.42% | 10480.98 | 10.16 |
| 180 | A0A091DLR0 | Uncharacterized protein | H | 0.77% | 171980.38 | 8.68 |
| 181 | A0A091DNL1 | Helicase ARIP4 | P | 0.75% | 162830.92 | 5.85 |
| 182 | A0A091DU44 | Inward rectifier potassium channel 2 | H | 1.39% | 87184.71 | 8.59 |
| 183 | A0A091DXX7 | Tumor necrosis factor receptor superfamily member 13B | P | 4.27% | 23367.59 | 8.76 |
| 184 | A0A091DZE3 | Thymidine kinase | H | 6.47% | 25353.3 | 8.79 |
| 185 | A0A091E7F1 | ATP synthase subunit g, mitochondrial | H | 6.80% | 11391.34 | 9.79 |
| 186 | A0A091E9G0 | Methenyltetrahydrofolate synthase domain-containing protein | H | 2.09% | 45833.96 | 6.94 |
| 187 | H0VH85 | Uncharacterized protein | H | 0.14% | 827538.32 | 5.29 |
| 188 | A0A091EF41 | Uncharacterized protein | P | 6.99% | 15613.65 | 4.89 |
| 189 | A0A091EFC1 | Histone-lysine N-methyltransferase | H | 0.22% | 300327.46 | 8.38 |
| 190 | A0A0A6YVR0 | Cytochrome c oxidase subunit 5B, mitochondrial | H | 11.22% | 10216.68 | 9.03 |
| 191 | A0A0A6YVW2 | DDB1- and CUL4-associated factor 6 | P | 5.30% | 15957.87 | 4.7 |
| 192 | A0A0C5PJP7 | HBB protein | P | 10.88% | 15906.18 | 7.92 |
| 193 | A0PK70 | Olfr661 protein | H | 4.70% | 35734.47 | 8.53 |
| 194 | A2A6Z2 | CMRF35-like molecule 1 | H | 2.70% | 32319.4 | 5.52 |
| 195 | Q0PKT2 | MHC class Ib antigen | P | 4.31% | 39062.71 | 5.97 |
| 196 | Q543Y2 | uncharacterized protein | P | 1.54% | 35262.36 | 5.09 |
| 197 | D3YV31 | Lysine-specific demethylase 2B | P | 3.15% | 33153.58 | 4.74 |
| 198 | Q8BM65 | Neuronal tyrosine-phosphorylated phosphoinositide-3-kinase adapter 2 | P | 0.88% | 73895.74 | 8.94 |
| 199 | D4A425 | Caspase recruitment domain family, member 10 （Predicted） | P | 1.76% | 114448.77 | 5.79 |
| 200 | D4A466 | Protein RGD1563307 | H | 2.56% | 31564.42 | 4.33 |
| 201 | Q80UK1 | Kinesin-like protein | P | 2.70% | 45413.77 | 7.12 |
| 202 | D4A891 | Protein Ccdc83 （Fragment） | H | 3.41% | 34491.79 | 4.86 |
| 203 | D4ABD7 | Protein Trip11 | H | 0.35% | 226328.1 | 5.27 |
| 204 | D4AC36 | Eukaryotic translation initiation factor 3, subunit 5 （Epsilon） （Predicted）, isoform CRA_a | P | 6.28% | 24549.5 | 5.87 |
| 205 | D9I2H0 | NLR family protein 1 | H | 0.66% | 138233.28 | 6.07 |
| 206 | Q3V5L4 | Tenascin-X | H | 0.51% | 339949.24 | 5.23 |
| 207 | Q5XI78 | 2-oxoglutarate dehydrogenase, mitochondrial | H | 0.88% | 116294.43 | 6.3 |
| 208 | F1LV80 | Protein Dnhd1 | H | 2.92% | 32177.45 | 5.64 |
| 209 | F1M614 | Protein Lama2 | H | 0.51% | 280617.01 | 5.64 |
| 210 | A0A023IKF6 | RT1-A1 | P | 1.78% | 38010.06 | 5.09 |
| 211 | I3N6S1 | Uncharacterized protein | H | 7.62% | 34118.25 | 7.27 |
| 212 | G3GUC0 | Rap guanine nucleotide exchange factor 4 | P | 1.58% | 86735.94 | 6.04 |
| 213 | P20717 | Protein-arginine deiminase type-2 | P | 1.95% | 75355.57 | 5.32 |
| 214 | G3GUD1 | Spermatogenesis-associated protein 7-like | H | 2.98% | 37800.67 | 9.67 |
| 215 | G3HAC6 | Titin | P | 0.03% | 4007027.71 | 5.75 |
| 216 | D3ZJ17 | Uncharacterized protein | H | 1.34% | 94135.37 | 9.41 |
| 217 | Q64033 | Antigen LEC-A | P | 1.00% | 67364.29 | 9.16 |
| 218 | Q9Z2Q1 | Protein transport protein Sec31A | P | 1.04% | 135270.49 | 6.65 |
| 219 | I3M1Z0 | Uncharacterized protein | P | 0.29% | 423218.43 | 6.52 |
| 220 | H0VKS2 | Cytochrome b-c1 complex subunit 7 | P | 8.11% | 13624.42 | 9.07 |
| 221 | G5BFV3 | FACT complex subunit SPT16 | P | 0.67% | 119971.45 | 5.5 |
| 222 | G5BGS2 | 60S ribosomal protein L23a | P | 8.33% | 9186.55 | 7.75 |
| 223 | G5BZ78 | UDP-glucuronosyltransferase 2B5 | H | 2.64% | 61337.99 | 8.34 |
| 224 | Q6P4T2 | U5 small nuclear ribonucleoprotein 200 kDa helicase | P | 0.33% | 244543.78 | 5.73 |
| 225 | G5BTE1 | AP-2 complex subunit mu-1 | H | 1.97% | 52003.62 | 9.65 |
| 226 | D3YXT0 | NADH dehydrogenase [ubiquinone] iron-sulfur protein 2, mitochondrial | H | 1.83% | 49572.74 | 8.34 |
| 227 | G5C0D9 | Junctional adhesion molecule C | H | 1.83% | 36761.7 | 7.55 |
| 228 | I3MNQ5 | Uncharacterized protein | H | 1.66% | 104783.39 | 6.2 |
| 229 | A0A091CP27 | Acetyltransferase component of pyruvate dehydrogenase complex | P | 2.30% | 69419.48 | 8.53 |
| 230 | G9BNN5 | Interferon gamma | P | 6.40% | 19367.01 | 8.56 |
| 231 | H0UU94 | Uncharacterized protein | H | 0.56% | 204726.46 | 5.53 |
| 232 | H0UUU7 | Uncharacterized protein | P | 0.29% | 393183.76 | 5.3 |
| 233 | H0UVS7 | Uncharacterized protein | P | 2.73% | 65415.53 | 6.73 |
| 234 | H0UZG1 | Tyrosine-protein kinase receptor | P | 0.82% | 91195.18 | 8.06 |
| 235 | H0UZQ8 | Uncharacterized protein | H | 1.29% | 71359.62 | 5.3 |
| 236 | H0V0L3 | Ubiquitin carboxyl-terminal hydrolase | P | 0.81% | 138850.62 | 5.55 |
| 237 | H0V7F0 | Uncharacterized protein | H | 3.16% | 43456.1 | 5.75 |
| 238 | H0V8B5 | Uncharacterized protein | P | 0.65% | 266180.06 | 5.28 |
| 239 | H0VBQ5 | Uncharacterized protein | P | 0.57% | 233610.67 | 6.19 |
| 240 | H0VC73 | Uncharacterized protein | P | 7.22% | 20484.77 | 4.23 |
| 241 | H0VLH4 | Uncharacterized protein | H | 7.89% | 12512.7 | 9.77 |
| 242 | H0VPE8 | Uncharacterized protein | P | 0.70% | 123480.21 | 5.85 |
| 243 | H0VQ95 | Uncharacterized protein | P | 0.32% | 208535.07 | 6.45 |
| 244 | H0VTI6 | Uncharacterized protein | H | 0.95% | 81558.25 | 6.68 |
| 245 | H0VXJ5 | Uncharacterized protein | P | 1.00% | 78357.31 | 8.6 |
| 246 | H0VYY3 | Uncharacterized protein | P | 1.62% | 62686.86 | 6.93 |
| 247 | H0W1H8 | Uncharacterized protein | P | 4.80% | 29977.09 | 8.46 |
| 248 | H0W4D8 | Uncharacterized protein | P | 0.78% | 237522.04 | 6.38 |
| 249 | I3N8X5 | Uncharacterized protein | H | 0.46% | 289460.26 | 7.55 |
| 250 | H0WB65 | Uncharacterized protein | P | 0.78% | 110018.41 | 4.93 |
| 251 | H0WBJ0 | Uncharacterized protein | P | 2.16% | 42869.67 | 5.7 |
| 252 | H0WD30 | Uncharacterized protein | H | 4.42% | 38381.87 | 9.79 |
| 253 | I3LX70 | Annexin | H | 3.45% | 35909.44 | 5.5 |
| 254 | I3M100 | Isocitrate dehydrogenase [NADP] | H | 1.11% | 50841.58 | 8.79 |
| 255 | I3M1E9 | Uncharacterized protein | P | 2.28% | 35112.42 | 5.49 |
| 256 | I3M2P3 | uncharacterized protein | P | 1.68% | 60223.89 | 5.59 |
| 257 | I3M5U2 | Uncharacterized protein | H | 2.99% | 30712 | 9.93 |
| 258 | I3MB63 | Uncharacterized protein | H | 3.98% | 36342.81 | 9.06 |
| 259 | I3MC38 | Uncharacterized protein | P | 0.99% | 107957.11 | 6.05 |
| 260 | I3MEL2 | Uncharacterized protein | H | 1.39% | 81349.02 | 10.33 |
| 261 | I3MHG3 | Uncharacterized protein | H | 5.24% | 23943.12 | 6.13 |
| 262 | I3MHU4 | Uncharacterized protein | H | 0.94% | 118458.52 | 9.35 |
| 263 | I3MKK5 | Uncharacterized protein | P | 2.24% | 46528.61 | 6.31 |
| 264 | I3MMZ2 | Uncharacterized protein | P | 3.53% | 35624.16 | 9.13 |
| 265 | A0A091CYC3 | Proteasome subunit beta type | P | 4.60% | 25550.8 | 4.99 |
| 266 | I3MQS1 | Uncharacterized protein | P | 3.69% | 26288.15 | 5.67 |
| 267 | I3MUU7 | Uncharacterized protein | H | 1.02% | 110939.63 | 6.68 |
| 268 | I3MW71 | Uncharacterized protein | P | 0.85% | 143566.09 | 6.29 |
| 269 | H0VLJ0 | Uncharacterized protein | H | 9.70% | 15487.19 | 4.45 |
| 270 | I3MWZ5 | Uncharacterized protein | H | 2.01% | 50681.1 | 7.95 |
| 271 | I3MYG1 | Uncharacterized protein | P | 15.85% | 9698.08 | 10.16 |
| 272 | I3MYW8 | Uncharacterized protein | H | 1.43% | 72043.73 | 8.61 |
| 273 | I3N9I4 | Uncharacterized protein | P | 3.78% | 27917.42 | 9.21 |
| 274 | I3NCR6 | Uncharacterized protein | P | 0.63% | 144817.25 | 7.7 |
| 275 | I3NDJ1 | Kinesin-like protein | P | 0.24% | 499243.22 | 6.01 |
| 276 | I3NDU2 | Uncharacterized protein | H | 3.61% | 50529.5 | 4.7 |
| 277 | I3NH29 | Uncharacterized protein | H | 0.75% | 105100.2 | 6.48 |
| 278 | A0A509 | MCG4625 | P | 2.22% | 55315.66 | 9.02 |
| 279 | M0R7M4 | Protein Rad51ap2 | P | 0.62% | 111116.51 | 8.04 |
| 280 | H0VHL2 | Uncharacterized protein | H | 1.41% | 47159.81 | 7.55 |
| 281 | O88483 | [Pyruvate dehydrogenase [acetyl-transferring]]-phosphatase 1, mitochondrial | H | 1.67% | 61206.62 | 6.34 |
| 282 | P08852 | Hemoglobin subunit alpha | P | 2.58% | 37572.3 | 6.75 |
| 283 | H0V777 | Uncharacterized protein | P | 5.31% | 33649.45 | 7.76 |
| 284 | P15178 | Aspartate--tRNA ligase, cytoplasmic | P | 2.40% | 57125.57 | 6.02 |
| 285 | P47757 | F-actin-capping protein subunit beta | P | 3.25% | 31345.12 | 5.47 |
| 286 | P15650 | Long-chain specific acyl-CoA dehydrogenase, mitochondrial | H | 2.33% | 47872.31 | 7.63 |
| 287 | G5BEH9 | Ribose-phosphate pyrophosphokinase 2 | P | 5.26% | 35190.27 | 6.08 |
| 288 | P48721 | Stress-70 protein, mitochondrial | H | 2.36% | 73856.85 | 5.97 |
| 289 | Q0QAL5 | Cytochrome b | P | 13.04% | 7478.76 | 8.17 |
| 290 | Q2LKW6 | NACHT-, LRR-, and PYD-containing protein 1 paralog b | H | 0.65% | 140686.66 | 6.59 |
| 291 | H0VBB2 | Uncharacterized protein OS=Cavia porcellus | H | 1.61% | 73860.41 | 5.94 |
| 292 | Q80WC7 | Arf-GAP domain and FG repeat-containing protein 2 | H | 3.97% | 48967.46 | 9.27 |
| 293 | P86207 | Ras-related protein Rab-2A | P | 21.67% | 6688.54 | 5.66 |
| 294 | Q3TJH1 | Putative uncharacterized protein | H | 2.82% | 40538.69 | 5.41 |
| 295 | P47753 | F-actin-capping protein subunit alpha-1 | P | 3.50% | 32939.35 | 5.34 |
| 296 | Q3U2F0 | Putative uncharacterized protein | H | 2.99% | 26245.39 | 5.63 |
| 297 | Q9D668 | Arrestin domain-containing protein 2 | P | 4.42% | 44258.34 | 9.32 |
| 298 | Q3UNH4 | protein-regulated inducer of neurite outgrowth 1 | P | 1.50% | 95494.84 | 8.14 |
| 299 | Q542D6 | Serine palmitoyltransferase, long chain base subunit 2, isoform CRA_a | P | 1.25% | 62980.88 | 8.43 |
| 300 | I3NG70 | Uncharacterized protein | H | 2.31% | 58082.5 | 4.93 |
| 301 | Q7TMS4 | Mpo protein OS=Mus musculus | P | 1.39% | 81166.73 | 9.63 |
| 302 | P07724 | Serum albumin | P | 2.14% | 68691.82 | 5.75 |
| 303 | Q5FVC4 | DnaJ （Hsp40） homolog, subfamily B, member 12 | P | 2.91% | 42520.88 | 8.64 |
| 304 | Q5XI57 | Protein polyglycylase TTLL10 | H | 2.21% | 77080.1 | 9.55 |
| 305 | Q14V05 | Tat-binding protein-1 | P | 5.52% | 20344.1 | 5.54 |
| 306 | I3MUN3 | Succinyl-CoA ligase subunit beta | P | 2.80% | 38443.89 | 5.52 |
| 307 | A4FUV6 | Met protein | P | 0.44% | 153458.74 | 6.62 |
| 308 | B5DFI6 | Rnf31 protein （Fragment） | H | 4.86% | 59237.03 | 7.51 |
| 309 | Q76LW6 | DAN domain family member 5 | P | 7.03% | 19771.7 | 9.77 |
| 310 | I3LXZ1 | Uncharacterized protein | P | 1.73% | 64083.51 | 4.99 |
| 311 | Q80X02 | HLC （Fragment） | P | 9.23% | 7392.18 | 5.46 |
| 312 | Q8BTI8 | Serine/arginine repetitive matrix protein 2 | P | 0.55% | 294836.91 | 12.02 |
| 313 | Q8K4E0 | Alstrom syndrome protein 1 homolog | H | 0.91% | 107667.54 | 4.77 |
| 314 | Q8VCI7 | ATPase SWSAP1 | P | 5.40% | 29384.22 | 5.44 |
| 315 | Q9ESW0 | DNA damage-binding protein 1 | P | 1.32% | 126860.45 | 5.21 |
| 316 | A0A0A0MY10 | Alsin | P | 0.67% | 182439.92 | 5.91 |
| 317 | Q3USD5 | Phosphate cytidylyltransferase 2, ethanolamine, isoform CRA_a | P | 2.97% | 45234.46 | 6.11 |
| 318 | Q99N89 | 39S ribosomal protein L43, mitochondrial | P | 6.56% | 20202.53 | 9.59 |
| 319 | Q9CUW8 | Putative uncharacterized protein | P | 2.62% | 21765.74 | 9.8 |
| 320 | H0VN45 | Uncharacterized protein | H | 0.92% | 87620.85 | 5.51 |
| 321 | Q9D8T2 | Gasdermin-D | P | 2.05% | 53237.36 | 5.03 |
| 322 | Q8CHT0 | Delta-1-pyrroline-5-carboxylate dehydrogenase, mitochondrial | H | 1.78% | 61839.82 | 8.45 |
| 323 | D3YVD3 | Gamma-enolase | H | 14.88% | 13265 | 5.06 |
| 324 | G3HR00 | NADH dehydrogenase [ubiquinone] 1 alpha subcomplex subunit 4 | H | 9.84% | 14152.76 | 10.02 |
| 325 | G5BXR0 | Acetyl-CoA acetyltransferase, mitochondrial | H | 3.16% | 43315.89 | 9.09 |
| 326 | P13601 | Aldehyde dehydrogenase, cytosolic 1 | H | 2.00% | 54559.04 | 7.1 |

SAα2-3Gal: sialic acid α2-3 galactose; MAL-II: *Maackia amurensis* lectin II; P: Pre-hibernation; H: Hibernation.
