# Supplementary material for: Remarkable Homeostasis of Protein Sialylation in Skeletal Muscles of Hibernating Daurian Ground Squirrels (Spermophilus dauricus)
Source: Front Physiol. 2020 Feb 7;11:37. doi: 10.3389/fphys.2020.00037 (PMC7020753; doi:10.3389/fphys.2020.00037)
Supplement: TABLE S3 — Significantly enriched KEGG pathways of glycoproteins with the SAα2-3Gal structure in the SOL muscles of Daurian ground squirrels in the PRE group (PDF, 75 kb). [file Table_3.DOCX]

**Table S3. Significantly enriched KEGG pathways of glycoproteins with the SAα2-3Gal structure in the SOL muscle of Daurian ground squirrels in the PRE group.**

| No. | Pathway ID | Pathway Name | Protein Count | P-Value |
| --- | --- | --- | --- | --- |
| 1 | ko04530 | Tight junction | 7 | 0.0002 |
| 2 | ko04260 | Cardiac muscle contraction | 8 | 2.48E-05 |
| 3 | ko00190 | Oxidative phosphorylation | 8 | 0.0004 |
| 4 | ko05010 | Alzheimer's disease | 16 | 3.99E-10 |
| 5 | ko05012 | Parkinson's disease | 14 | 5.87E-09 |
| 6 | ko05016 | Huntington's disease | 13 | 1.16E-07 |
| 7 | ko04145 | Phagosome | 6 | 0.0106 |
| 8 | ko04510 | Focal adhesion | 5 | 0.0230 |
| 9 | ko05130 | Pathogenic Escherichia coli infection | 4 | 0.0009 |
| 10 | ko05410 | Hypertrophic cardiomyopathy （HCM） | 4 | 0.0087 |
| 11 | ko05412 | Arrhythmogenic right ventricular cardiomyopathy | 3 | 0.0342 |
| 12 | ko05414 | Dilated cardiomyopathy | 4 | 0.0104 |
| 13 | ko00500 | Starch and sucrose metabolism | 3 | 0.0181 |
| 14 | ko00944 | Flavone and flavonol biosynthesis | 1 | 0.0132 |
| 15 | ko05169 | Epstein-Barr virus infection | 5 | 0.0373 |
| 16 | ko04932 | Non-alcoholic fatty liver disease （NAFLD） | 14 | 2.4E-09 |
| 17 | ko00062 | Fatty acid elongation | 2 | 0.0169 |
| 18 | ko00071 | Fatty acid degradation | 4 | 0.0010 |
| 19 | ko00280 | Valine, leucine and isoleucine degradation | 4 | 0.0014 |
| 20 | ko00362 | Benzoate degradation | 2 | 0.0023 |
| 21 | ko01212 | Fatty acid metabolism | 4 | 0.0011 |
| 22 | ko00010 | Glycolysis / Gluconeogenesis | 3 | 0.0277 |
| 23 | ko00710 | Carbon fixation in photosynthetic organisms | 2 | 0.0221 |
| 24 | ko01200 | Carbon metabolism | 10 | 1.42E-07 |
| 25 | ko01230 | Biosynthesis of amino acids | 5 | 0.0009 |
| 26 | ko04066 | HIF-1 signaling pathway | 4 | 0.0211 |
| 27 | ko04540 | Gap junction | 4 | 0.0094 |
| 28 | ko04974 | Protein digestion and absorption | 3 | 0.0328 |
| 29 | ko04972 | Pancreatic secretion | 4 | 0.0186 |
| 30 | ko00020 | Citrate cycle （TCA cycle） | 4 | 0.0003 |
| 31 | ko00720 | Carbon fixation pathways in prokaryotes | 2 | 0.0074 |
| 32 | ko00620 | Pyruvate metabolism | 3 | 0.0059 |
| 33 | ko05211 | Renal cell carcinoma | 3 | 0.0192 |
| 34 | ko00680 | Methane metabolism | 2 | 0.0255 |
| 35 | ko05143 | African trypanosomiasis | 2 | 0.0446 |
| 36 | ko00030 | Pentose phosphate pathway | 2 | 0.0243 |
| 37 | ko03050 | Proteasome | 3 | 0.0084 |
| 38 | ko00650 | Butanoate metabolism | 2 | 0.0178 |
| 39 | ko05014 | Amyotrophic lateral sclerosis （ALS） | 3 | 0.0187 |
| 40 | ko00640 | Propanoate metabolism | 2 | 0.0243 |

SAα2-3Gal: sialic acid α2-3 galactose; PRE: pre-hibernation group.
