# Supplementary material for: Remarkable Homeostasis of Protein Sialylation in Skeletal Muscles of Hibernating Daurian Ground Squirrels (Spermophilus dauricus)
Source: Front Physiol. 2020 Feb 7;11:37. doi: 10.3389/fphys.2020.00037 (PMC7020753; doi:10.3389/fphys.2020.00037)
Supplement: TABLE S4 — Significantly enriched KEGG pathways of glycoproteins with the SAα2-3Gal structure in the SOL muscles of Daurian ground squirrels in the HIB group (PDF, 72 kb). [file Table_4.DOCX]

**Table S4. Significantly enriched KEGG pathways of glycoproteins with the SAα2-3Gal structure in the SOL muscle of Daurian ground squirrels in the HIB group.**

| No. | Pathway ID | Pathway Name | Protein Count | P-Value |
| --- | --- | --- | --- | --- |
| 1 | ko04530 | Tight junction | 6 | 0.000381 |
| 2 | ko00190 | Oxidative phosphorylation | 14 | 2.34E-10 |
| 3 | ko05010 | Alzheimer's disease | 17 | 1.56E-12 |
| 4 | ko05012 | Parkinson's disease | 15 | 3.51E-11 |
| 5 | ko05016 | Huntington's disease | 15 | 1.01E-10 |
| 6 | ko00500 | Starch and sucrose metabolism | 3 | 0.010728 |
| 7 | ko04922 | Glucagon signaling pathway | 4 | 0.005252 |
| 8 | ko04260 | Cardiac muscle contraction | 7 | 4.96E-05 |
| 9 | ko05416 | Viral myocarditis | 4 | 0.007947 |
| 10 | ko05410 | Hypertrophic cardiomyopathy （HCM） | 5 | 0.000574 |
| 11 | ko05414 | Dilated cardiomyopathy | 5 | 0.000726 |
| 12 | ko04510 | Focal adhesion | 5 | 0.01481 |
| 13 | ko05130 | Pathogenic Escherichia coli infection | 2 | 0.042837 |
| 14 | ko05132 | Salmonella infection | 3 | 0.023151 |
| 15 | ko05164 | Influenza A | 6 | 0.001848 |
| 16 | ko05412 | Arrhythmogenic right ventricular cardiomyopathy | 3 | 0.020904 |
| 17 | ko00010 | Glycolysis / Gluconeogenesis | 5 | 0.000245 |
| 18 | ko01200 | Carbon metabolism | 11 | 1.44E-09 |
| 19 | ko01230 | Biosynthesis of amino acids | 5 | 0.000341 |
| 20 | ko00620 | Pyruvate metabolism | 5 | 1.37E-05 |
| 21 | ko05230 | Central carbon metabolism in cancer | 3 | 0.013036 |
| 22 | ko00062 | Fatty acid elongation | 2 | 0.011486 |
| 23 | ko00071 | Fatty acid degradation | 6 | 1.78E-06 |
| 24 | ko00280 | Valine, leucine and isoleucine degradation | 5 | 4.81E-05 |
| 25 | ko00362 | Benzoate degradation | 2 | 0.001512 |
| 26 | ko01212 | Fatty acid metabolism | 6 | 2.11E-06 |
| 27 | ko04141 | Protein processing in endoplasmic reticulum | 4 | 0.020629 |
| 28 | ko05134 | Legionellosis | 3 | 0.008658 |
| 29 | ko05145 | Toxoplasmosis | 6 | 0.00049 |
| 30 | ko05169 | Epstein-Barr virus infection | 5 | 0.018952 |
| 31 | ko04932 | Non-alcoholic fatty liver disease （NAFLD） | 11 | 1.7E-07 |
| 32 | ko04972 | Pancreatic secretion | 4 | 0.009748 |
| 33 | ko04540 | Gap junction | 3 | 0.027961 |
| 34 | ko00020 | Citrate cycle （TCA cycle） | 7 | 9.35E-09 |
| 35 | ko04512 | ECM-receptor interaction | 3 | 0.037654 |
| 36 | ko05020 | Prion diseases | 2 | 0.039576 |

SAα2-3Gal: sialic acid α2-3 galactose; HIB: hibernation group.
